# Supplementary material for: A tiny loop in the Argonaute PIWI domain tunes small RNA seed strength
Source: EMBO Rep. 2023 Apr 21;24(6):e55806. doi: 10.15252/embr.202255806 (PMC10240194; doi:10.15252/embr.202255806)
Supplement: Supplementary file 1 — Expanded View Figures PDF [file EMBR-24-e55806-s002.pdf]

Expanded View Figures

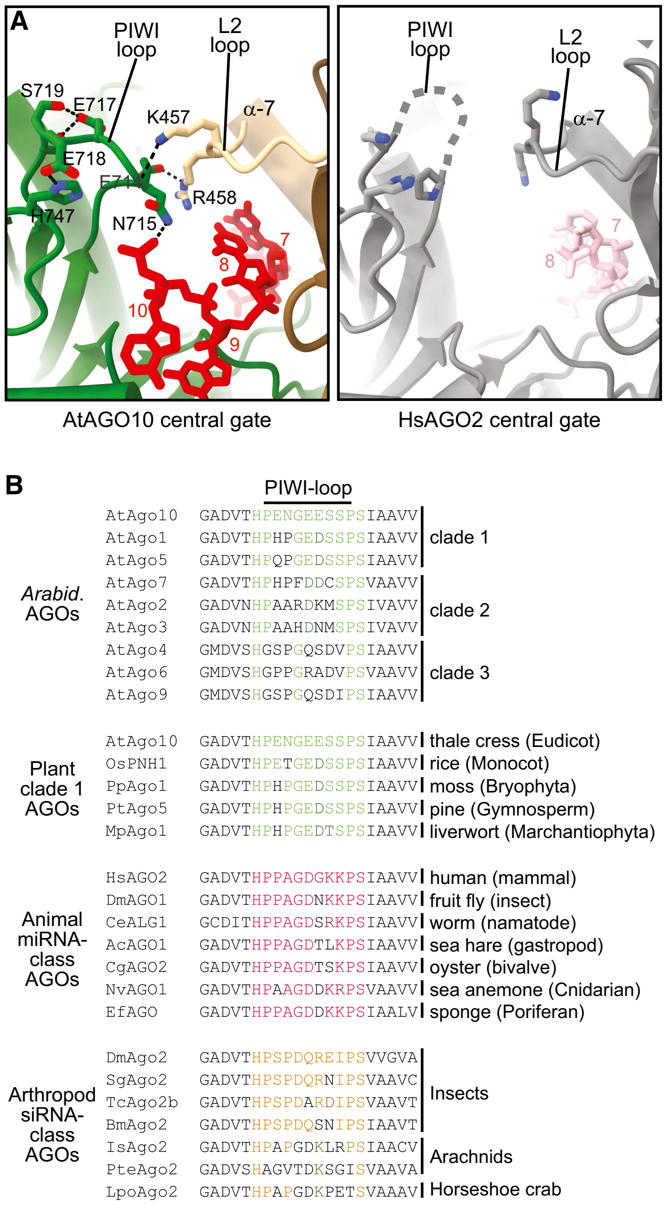

Figure EV1. Comparison of PIWI loops in diverse AGOs.

A Close-up views of PIWI-loop and L2-loop connections in AtAGO10 (PDB 7SVA) and HsAGO2 (PDB 4OLA) structures.

B Alignment of PIWI-loop sequences from diverse eukaryotes.

© 2023 The Authors

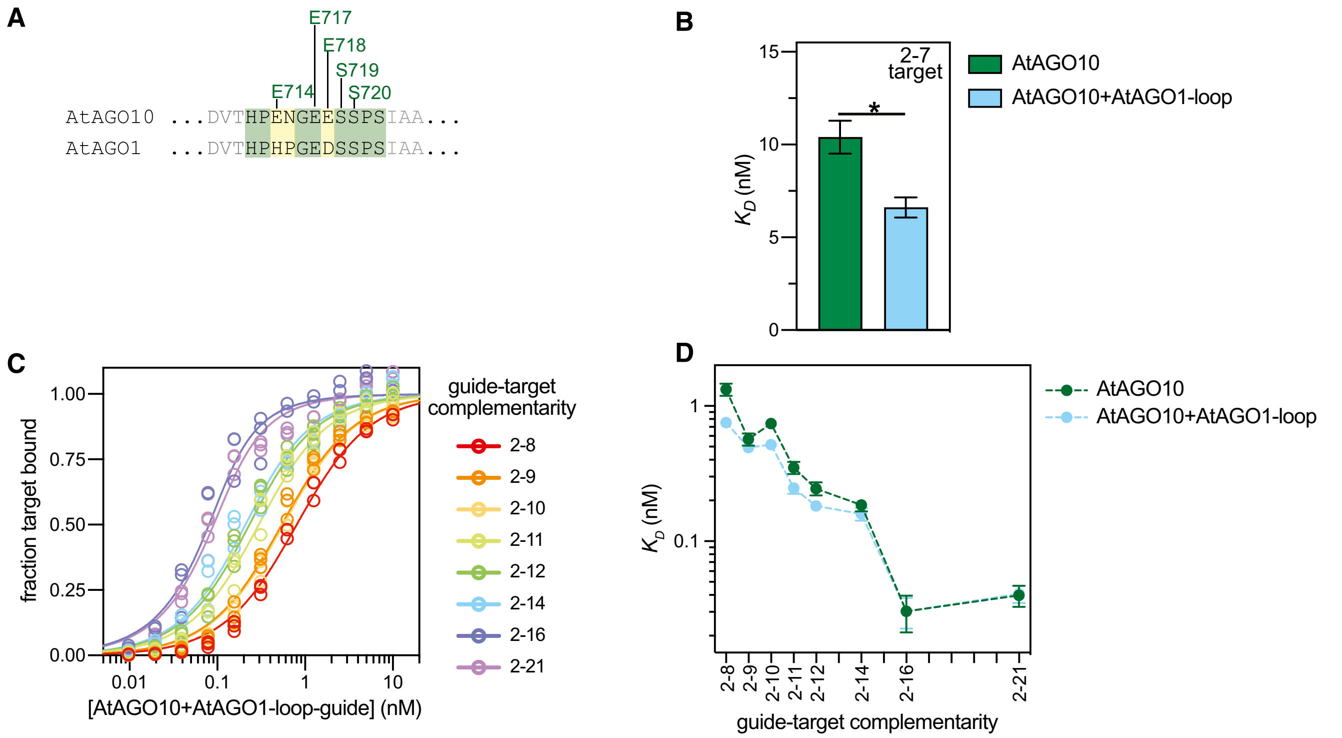

Figure EV2. PIWI loops of AtAGO1 and AtAGO10 are functionally similar.

A Detailed comparison of PIWI-loop sequences in AtAGO10 and AtAGO1.

B Dissociation constants for wild-type AtAGO10 and a mutant AtAGO10 bearing the PIWI-loop sequence from AtAGO1 (AtAGO10 + AtAGO10-loop) binding to a target RNA with complementarity to the miRNA seed region (2–7).

C Fraction target RNAs bound versus [AtAGO1 + AtAGO1-loop]. Guide and target RNAs are shown in Fig 2A.

D K_D values of wild-type AtAGO10 and AtAGO10 + AtAGO1-loop mutant for various target RNAs.

Data information: Data points in panel (C) are from $n = 3$ independent technical trials. Data in panels (B and D) represent best-fit K_D values from $n = 3$ technical trials. Error bars indicate s.e.m. Unpaired t-test P-value: *0.0219. No data points were excluded from the analyses.

© 2023 The Authors

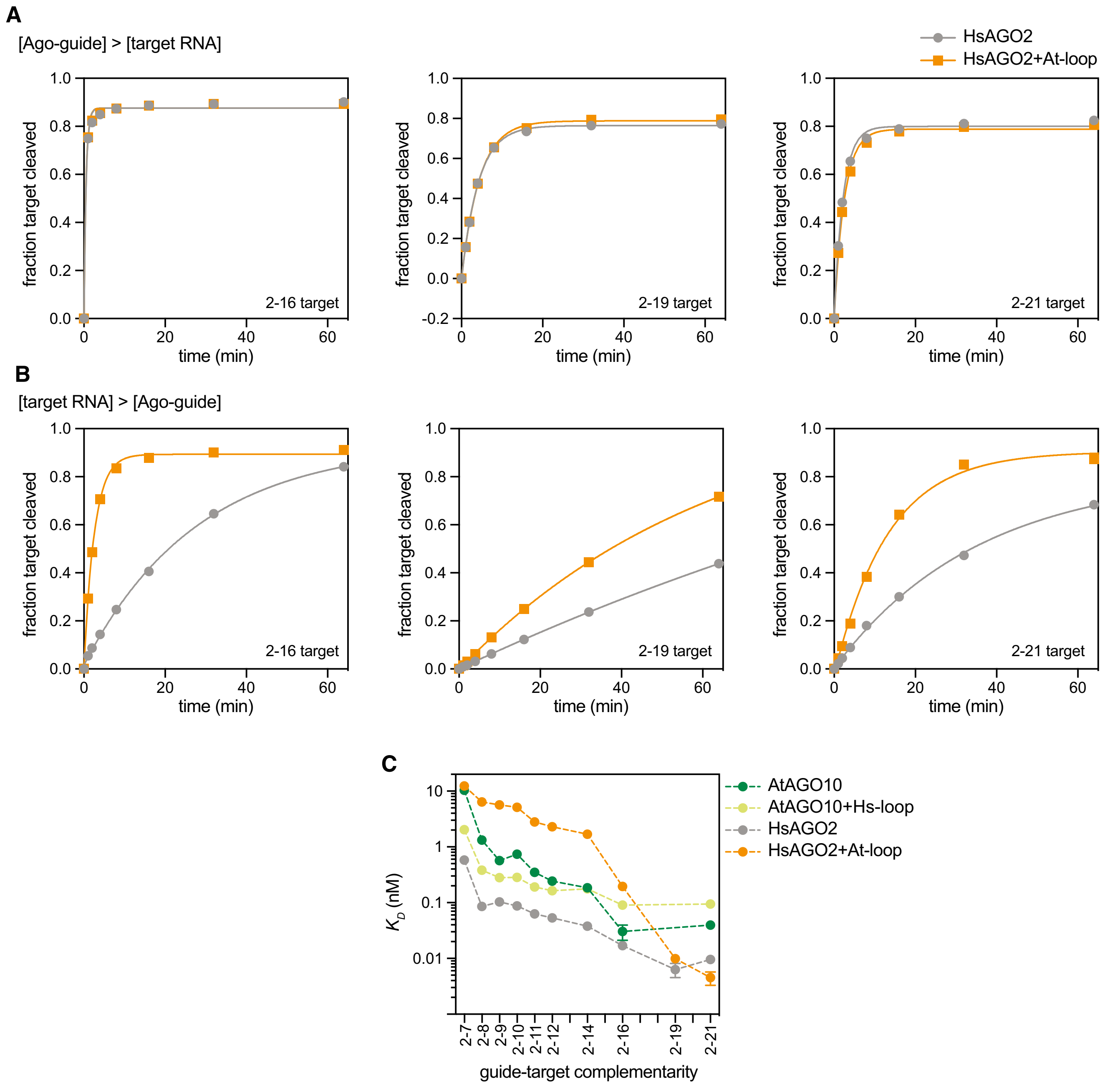

Figure EV3. Detailed comparison of HsAGO2 and HsAGO2 + At-loop biochemical properties.

A Fraction of target RNAs cleaved versus time when cleavage reaction is limited by target RNA ([Ago-guide] = 10 nM, [target RNA] = 2 nM).
B Fraction of target RNAs cleaved versus time when cleavage reaction is limited by Ago-guide ([Ago-guide] = 1 nM, [target RNA] = 5 nM).
C Comparison of K_D values of HsAGO2, HsAGO2 + At-loop, AtAGO10, and AtAGO10 + Hs-loop for various target RNAs.

Data information: Data points in panels (A and B) are values from $n = 1$ experimental trials. Data points in panel (C) are the best-fit K_D values from $n = 3$ independent technical trials, each with ≥ 11 data points. Error bars indicate s.e.m. No data points were excluded from the analyses.

© 2023 The Authors
