# Supplementary material for: A tiny loop in the Argonaute PIWI domain tunes small RNA seed strength
Source: EMBO Rep. 2023 Apr 21;24(6):e55806. doi: 10.15252/embr.202255806 (PMC10240194; doi:10.15252/embr.202255806)
Supplement: Supplementary file 2 — Table EV1 [file EMBR-24-e55806-s005.docx]

|  | **AtAGO10** | **AtAGO10+**  **AtAGO1-loop** | **AtAGO10+Hs-loop** | **HsAGO2** | **HsAGO2+At-loop** |
| --- | --- | --- | --- | --- | --- |
| ***K_D_* values (pM)** |  |  |  |  |  |
|  |  |  |  |  |  |
| 2–8 target | 1330 ± 140 | 753 ± 46 | 383 ± 31 | 86.2 ± 4.7 | 6430 ± 240 |
| 2–9 target | 568 ± 58 | 492 ± 36 | 282 ± 26 | 103 ± 5.9 | 5680 ± 250 |
| 2–10 target | 740 ± 65 | 518 ± 36 | 283 ± 23 | 87.9 ± 5.3 | 5160 ± 320 |
| 2–11 target | 350 ± 36 | 247 ± 22 | 192 ± 18 | 63.2 ± 4.0 | 2800 ± 120 |
| 2–12 target | 245 ± 27 | 182 ± 14 | 164 ± 15 | 53.2 ± 3.3 | 2300 ± 85 |
| 2–14 target | 185 ± 19 | 159 ± 17 | 177 ± 19 | 38.1 ± 2.9 | 1700 ± 55 |
| 2–16 target | 30.4 ± 9.2 | 30.4 ± 7.7 | 90.7 ± 12.2 | 17.1 ± 1.6 | 195 ± 9.4 |
| 2–19 target |  |  |  | 6.3 ± 1.8 | 9.9 ± 1.4 |
| 2–21 target | 39.8 ± 7.1 | 40.9 ± 6.0 | 95.0 ± 9.0 | 9.6 ± 1.3 | 4.5 ± 1.2 |
|  |  |  |  |  |  |
| ***k_off, slow_* rates**  **(min^-1^)** |  |  |  |  |  |
| 2–8 target | 0.116 ± 0.017 (17%) | 0.091 ± 0.006 (90%) |  |  |  |
| 2–9 target | 0.319 ± 0.014 (51%) | 0.098 ± 0.009 (94%) |  |  |  |
| 2–10 target | 0.375 ± 0.024 (42%) | 0.101 ± 0.010 (92%) |  |  |  |
| 2–11 target | 0.119 ± 0.010 (72%) | 0.026 ± 0.002 (96%) |  |  |  |
| 2–12 target | 0.099 ± 0.007 (85%) | 0.026 ± 0.002 (95%) |  |  |  |
| 2–14 target | 0.034 ± 0.002 (87%) | 0.016 ± 0.002 (96%) |  |  |  |
| 2–16 target | 0.008 ± 0.001 (100%) |  |  |  |  |
| 2–21 target | 0.012 ± 0.002 (100%) |  |  |  |  |
|  |  |  |  |  |  |
| **cleavage rate constants (s^-1^)** |  |  |  |  |  |
| *k_2_*, 22˚C |  |  |  | 0.162 ± 0.058 | 0.030 ± 0.014 |
| *k_2_*, 36 ˚C |  |  |  | 0.174 ± 0.135 | 0.110 ± 0.058 |
| *k_3_*, 22˚C |  |  |  | 0.0062 ± 0.0012 | 0.0088 ± 0.0043 |
| *k_3_*, 36 ˚C |  |  |  | 0.0148 ± 0.0029 | 0.0578 ± 0.0146 |

**Table EV1. Kinetic constants determined in this study.** Dissociation constants (*K_D_*) of AGO-guide complexes for target RNAs are indicated. Slow phase rates (*k_off,slow_*) of target release fit to a two-phase decay with a fast phase (*k_off,fast_* = 4.626 min^-1^) globally fit to all data. Parentheses indicate the percent of the slow phase in each data fit. First-order rate constants (*k_2_* and *k_3_*) for cleavage and release of the 2–19 target RNA at 22 ˚C or 36 ˚C. The standard error of the mean (SEM) of each value is indicated by ±.
