# Supplementary material for: A tiny loop in the Argonaute PIWI domain tunes small RNA seed strength
Source: EMBO Rep. 2023 Apr 21;24(6):e55806. doi: 10.15252/embr.202255806 (PMC10240194; doi:10.15252/embr.202255806)
Supplement: Supplementary file 3 — Table EV2 [file EMBR-24-e55806-s003.docx]

| **Structure** | **HsAGO2-miR122** | **HsAGO2+At-loop-miR122** |
| --- | --- | --- |
| PDB Code | 8D71 | 8D6J |
| Wavelength (Å) | 0.97946 | 0.97946 |
| Resolution range (Å) | 37.71 - 2.5 (2.589 - 2.5) | 37.69 - 2.5 (2.589 - 2.5) |
| Space group | P 1 2_1_ 1 | P 1 2_1_ 1 |
| Unit cell lengths (Å) | 63.13, 107.74, 68.82 | 63.30, 107.51, 68.71, |
| Unit cell angles (˚) | 90, 106.89, 90 | 90, 106.90, 90 |
| Total reflections | 60147 (5968) | 60166 (5980) |
| Unique reflections | 30149 (2990) | 30141 (2992) |
| Multiplicity | 2.0 (2.0) | 2.0 (2.0) |
| Completeness (%) | 98.72 (99.27) | 98.75 (99.17) |
| Mean I/sigma(I) | 23.81 (4.81) | 12.90 (2.58) |
| Wilson B-factor | 47.93 | 49.32 |
| R-merge | 0.01911 (0.1429) | 0.03704 (0.2961) |
| R-meas | 0.02702 (0.2021) | 0.05239 (0.4187) |
| R-pim | 0.01911 (0.1429) | 0.03704 (0.2961) |
| CC1/2 | 0.999 (0.956) | 0.998 (0.832) |
| CC* | 1 (0.989) | 0.999 (0.953) |
| Reflections used in refinement | 30144 (2990) | 30135 (2992) |
| Reflections used for R-free | 1224 (122) | 1313 (134) |
| R-work | 0.2242 (0.2756) | 0.2326 (0.2996) |
| R-free | 0.2800 (0.3314) | 0.2854 (0.3359) |
| CC (work) | 0.942 (0.856) | 0.937 (0.802) |
| CC (free) | 0.897 (0.796) | 0.871 (0.691) |
| No. of non-hydrogen atoms | 6780 | 6774 |
| macromolecules | 6716 | 6723 |
| ligands | 1 | 1 |
| Protein residues | 810 | 810 |
| RMSD bonds (Å) | 0.006 | 0.004 |
| RMSD angles (˚) | 0.61 | 0.58 |
| Ramachandran favored (%) | 94 | 93.86 |
| Ramachandran allowed (%) | 5.38 | 6.02 |
| Ramachandran outliers (%) | 0.62 | 0.13 |
| Rotamer outliers (%) | 0.97 | 1.11 |
| Clashscore | 4.86 | 7.1 |
| Average B-factor | 60.7 | 59.26 |
| macromolecules | 61.13 | 59.59 |
| ligands | 49.71 | 50.11 |

**Table EV2. Crystallographic and Refinement Statistics for human Ago2-miR-122 and human Ago2+At-loop-miR122 Complexes.** Numbers in parentheses represent statistics in the highest resolution shell. R_merge_ = (Σ_h_Σ_i_|I_h_ − I_hi_|/Σ_h_Σ_i_I_h,i_) × 100, where I_h_ is the mean of I_h,i_ observations of reflection h. R-pim = Σ_h_ [1/(/n_h_ - 1)]^1/2^ Σ_i_|<I_h_> I_h,i_|/Σ_h_ Σ_i_ I_h,i_ R-work and R-free = Σ||F_o_| − |F_c_||/Σ|F_o_| × 100 for 95% of recorded data (R-work) or 5% of data (R-free). RMSD, root-mean-square deviation. CC, correlation coefficient.
