# Supplementary material for: A tiny loop in the Argonaute PIWI domain tunes small RNA seed strength
Source: EMBO Rep. 2023 Apr 21;24(6):e55806. doi: 10.15252/embr.202255806 (PMC10240194; doi:10.15252/embr.202255806)
Supplement: Supplementary file 4 — Table EV3 [file EMBR-24-e55806-s006.docx]

| **Oligo name** | **Sequence (5' 🡪 3')** |
| --- | --- |
| AtAGO10+Hs-loop 5' frag forward primer | accgaaaacctgtattttcagggcATGCCGATTAGGCAAATGAAAG |
| AtAGO10+Hs-loop 5' frag reverse primer | cttcttcccatccccggcgggTGGGTGAGTCACGTCTGCGC |
| AtAGO10+Hs-loop 3' frag forward primer | CCAcccgccggggatgggaagaagCCTTCAATCGCTGCTGTTGTTG |
| AtAGO10+Hs-loop 3' frag reverse primer | ggccgcactagttgagctcgtcgacTTAGCAGTAGAACATTACTCTC |
| HsAGO2+At-loop 5' frag forward primer | accgaaaacctgtattttcagggcATGTACTCGGGAGCCGGCCC |
| HsAGO2+At-loop 5' frag reverse primer | GGGgcttgactcttccccgttctcGGGGTGAGTGACGTCTGCTCC |
| HsAGO2+At-loop 3' frag forward primer | gagaacggggaagagtcaagcCCCTCCATTGCCGCCGTGGTG |
| HsAGO2+At-loop 3' frag reverse primer | ggccgcactagttgagctcgtcgacTCAAGCAAAGTACATGGTGCGC |
| guide RNA | P-UGGAGUGUGACAAUGGUGUUU |
| 2–7 target RNA | AAAAAAAAAAAAACCACUCCAAA |
| 2–8 target RNA | AAAAAAAAAAAAAACACUCCAAA |
| 2–9 target RNA | AAAAAAAAAAAACACACUCCAAA |
| 2–10 target RNA | AAAAAAAAAAAUCACACUCCAAA |
| 2–11 target RNA | AAAAAAAAAAGUCACACUCCAAA |
| 2–12 target RNA | AAAAAAAAAUGUCACACUCCAAA |
| 2–14 target RNA | AAAAAAAAUUGUCACACUCCAAA |
| 2–16 target RNA | UUUUUUCCAUUGUCACACUCCAAA |
| 2–19 target RNA | UUACACCAUUGUCACACUCCAAA |
| 2–21 target RNA | AAACACCAUUGUCACACUCCAAA |

**Table EV3. Oligonucleotides used in this study.** Forward and reverse DNA primers used to generate AGO mutants are listed with capital letters indicating coding nucleotides of the ORF being mutated. P indicates a 5' phosphate on the guide RNA.
