# Supplementary material for: A tiny loop in the Argonaute PIWI domain tunes small RNA seed strength
Source: EMBO Rep. 2023 Apr 21;24(6):e55806. doi: 10.15252/embr.202255806 (PMC10240194; doi:10.15252/embr.202255806)
Supplement: Supplementary file 5 — PDF+ [file EMBR-24-e55806-s001.pdf]

Article

TRANSPARENT PROCESS · OPEN ACCESS

EMBO reports

A tiny loop in the Argonaute PIWI domain tunes small RNA seed strength

Yao Xiao, TingYu M Liu & Ian J MacRae*

Abstract

Argonaute (AGO) proteins use microRNAs (miRNAs) and small interfering RNAs (siRNAs) as guides to regulate gene expression in plants and animals. AGOs that use miRNAs in bilaterian animals recognize short (6–8 nt.) elements complementary to the miRNA seed region, enabling each miRNA to interact with hundreds of otherwise unrelated targets. By contrast, AGOs that use miRNAs in plants employ longer (> 13 nt.) recognition elements such that each miRNA silences a small number of physiologically related targets. Here, we show that this major functional distinction depends on a minor structural difference between plant and animal AGO proteins: a 9-amino acid loop in the PIWI domain. Swapping the PIWI loop from human Argonaute2 (HsAGO2) into *Arabidopsis* Argonaute10 (AtAGO10) increases seed strength, resulting in animal-like miRNA targeting. Conversely, swapping the plant PIWI loop into HsAGO2 reduces seed strength and accelerates the turnover of cleaved targets. The loop-swapped HsAGO2 silences targets more potently, with reduced miRNA-like targeting, than wild-type HsAGO2 in mammalian cells. Thus, tiny structural differences can tune the targeting properties of AGO proteins for distinct biological roles.

Keywords Argonaute; microRNA; miRNA; Piwi; siRNA
Subject Categories RNA Biology; Structural Biology
DOI 10.15252/embr.202255806 | Received 18 July 2022 | Revised 29 March 2023 | Accepted 5 April 2023 | Published online 21 April 2023
EMBO Reports (2023) 24: e55806

Introduction

AGO proteins use small RNA guides to identify complementary sites in transcripts targeted for silencing or repression. Overall, the three-dimensional structures of AGOs found in plants and animals are all similar to each other (preprint: Xiao *et al*, 2022). However, despite structural similarities, different AGOs often carry out distinct functions wherein the degree of guide complementarity necessary for target recognition and the molecular consequences of target recognition can vary between different AGO proteins.

In bilaterian animals, miRNA-class AGO proteins regulate the expression of target mRNAs (Bartel, 2018). miRNA-target recognition leads to a moderate (1.1–3 fold) reduction in gene expression via the recruitment of factors involved in translational repression and mRNA decay (Baek *et al*, 2008; Jonas & Izaurralde, 2015). The miRNA recognition elements (MREs) found in bilaterian mRNAs consist of a core segment of complementarity to miRNA guide (g) nucleotides g2–g7 (counting from the miRNA 5′ end), which is termed the miRNA seed region (Lewis *et al*, 2005). The short length (6–8 nt.) typical of animal MREs enables each miRNA to potentially interact with hundreds of different mRNAs (Friedman *et al*, 2009; Bartel, 2018), leading to the proposal that miRNAs function as sculptors of the transcriptome in metazoans (Bartel, 2018). Notably, physiological functions for most predicted miRNA-binding sites in mammals remain to be demonstrated. Taken with the moderate effect miRNA sites often have on target mRNA levels, it has alternatively been proposed that only a subset of miRNA-target interactions directly impact animal physiology while the majority of sites may function to titrate miRNA activity (Pinzon *et al*, 2017; Seitz, 2017).

Some animal AGOs are loaded with siRNAs, which are derived from long double-stranded RNAs, or can be delivered as synthetic small RNA duplexes (Elbashir *et al*, 2001a, 2001b). siRNA-mediated silencing involves extensive guide-target base pairing, which elicits an endonuclease activity in many AGO proteins. Nematodes and arthropods have been found to possess specialized siRNA-class AGOs that function as an antiviral defense (Wilkins *et al*, 2005; van Rij *et al*, 2006; Wang *et al*, 2006; Schnettler *et al*, 2014; Wynant *et al*, 2017). Vertebrates lack siRNA-class AGOs, but mammalian Argonaute2 (AGO2) can be loaded with siRNAs and cleave complementary targets (Liu *et al*, 2004; Meister *et al*, 2004; Rivas *et al*, 2005). Synthetic siRNAs are thus useful research tools for the transient knockdown of target genes in mammalian cell culture and have emerged in the clinic as a novel approach to treating human disease (Weng *et al*, 2019; Syed, 2021). However, because vertebrate AGO2 evolved primarily for miRNA-mediated repression (Cheloufi *et al*, 2010; Cifuentes *et al*, 2010; Chen *et al*, 2017), siRNAs in humans inevitably also function as miRNAs and can moderately repress unintended targets (Jackson & Linsley, 2010; Seok *et al*, 2018).

Plants produce miRNAs and several classes of siRNAs (Borges & Martienssen, 2015). Plants have an MRE core that is twice as long as typical mammalian MREs, comprising complementarity to g2–g13 (Allen *et al*, 2005). To our knowledge, unbiased mapping of AGO1/miRNA-binding sites throughout a plant transcriptome has not been reported. Thus, the extent to which plant miRNAs interact with target

Department of Integrative Structural and Computational Biology, The Scripps Research Institute, La Jolla, CA, USA
*Corresponding author. Tel: +1(858)784-2932; E-mail: macrae@scripps.edu

© 2023 The Authors. Published under the terms of the CC BY 4.0 license

mRNAs *in vivo* remains to be established. However, it is clear that the seed-based sites that function in animals do not reliably confer repression in plants (Liu *et al*, 2014). The requirement of extended miRNA-target complementarity for silencing in plants is explained, in part, by the observation that plant miRNAs often function through target RNA cleavage and thus act like siRNAs (Llave *et al*, 2002; Tang *et al*, 2003). However, plant miRNAs can also function through translational repression, without target RNA degradation (Aukerman & Sakai, 2003; Chen, 2004; Gandikota *et al*, 2007; Brodersen *et al*, 2008; Li *et al*, 2013), indicating that extensive miRNA-target complementarity may also be required for target recognition. Indeed, biochemical analysis of *Arabidopsis* AGO1 (AtAGO1) showed seed complementarity alone is insufficient for high-affinity target binding (Iwakawa & Tomari, 2013). Thus, miRNA-guided AGOs in plants appear to inherently require more guide-target complementarity for target recognition than AGOs in mammals.

We recently reported cryo-EM structures of *Arabidopsis* (At AGO10; preprint: Xiao *et al*, 2022). AtAGO10 has been classified into clade 1 of plant AGOs (Vaucheret, 2008). Clade 1 includes AtAGO1, the namesake of the AGO protein family (Bohmert *et al*, 1998), and is primarily responsible for miRNA function in plants (Vaucheret, 2008). Surprisingly, despite the functional distinctions between plant and animal miRNAs, AtAGO10 structure is remarkably similar to human AGOs (preprint: Xiao *et al*, 2022). Here, we explored the hypothesis that small structural differences between miRNA-guided AGOs in plants and animals are responsible for creating differential targeting properties. We identified a short loop in the PIWI domain of AtAGO10 that significantly influences targeting behavior. The loop is disordered in human AGO structures but forms a defined structure in AtAGO10. Structural analysis indicates the AtAGO10 loop decreases target affinity by stabilizing a closed AGO conformation that is incompatible with pairing to the 3′ half of the seed region. Swapping the loop from HsAGO2 into AtAGO10, and vice versa, produces an AtAGO10 variant with animal-like miRNA-targeting properties and an HsAGO2 variant with plant-like miRNA-targeting. The loop-swapped HsAGO2-siRNA complex has elevated siRNA potency and reduced miRNA-like targeting compared with conventional siRNAs and the wild-type HsAGO2-siRNA complex when introduced into mammalian cells. Combined results show that AGO-targeting behavior can be tuned by small alterations of the AGO primary sequence, explaining how the AGO protein family can mediate diverse physiological functions in eukaryotes and providing insights that may facilitate engineering HsAGO2 with enhanced therapeutic properties.

Results

Plant clade 1 AGOs create a weak guide RNA seed region

AGO proteins create the seed region by pre-organizing guide nucleotides g2–g7 in a helical conformation, thereby lowering the entropic cost of initiating target binding (Wang *et al*, 2008; Parker *et al*, 2009; Schirle & MacRae, 2012). In animal AGOs, the affinity of the seed region for targets is quite high, with dissociation constants typically in the subnanomolar range (Wee *et al*, 2012; Salomon *et al*, 2015; Jouravleva *et al*, 2022). Curiously, although AtAGO10 pre-organizes the seed region in a fashion identical to human AGOs (Fig 1A and B), AtAGO1 failed to bind target RNAs with complementary limited to the seed (Iwakawa & Tomari, 2013). These observations indicate plant clade 1 AGOs may dampen the affinity of the pre-organized seed through an unknown mechanism. To test this idea directly, we measured the affinity of purified the AtAGO10-miRNA complex for a seed-matched target RNA. Similar to results with AtAGO1 (Iwakawa & Tomari, 2013), AtAGO10 bound a seed-matched target with an 18-fold lower affinity than that of HsAGO2 using the same miRNA guide (Fig 1C; Table EV1). Thus, although miRNA-guided AGOs from plant and animals pre-organize the seed region in the same way, the plant AGOs possess a means of dampening seed strength.

A loop in the AtAGO10 PIWI domain dampens seed pairing

To understand how AtAGO10 dampens its seed region, we compared the AtAGO10-miRNA structure to human AGO-miRNA structures. There are multiple amino acid substitutions surrounding the seed region, but the largest difference, in terms of protein backbone conformation, is a PIWI domain loop immediately downstream of the seed (Figs 1B and EV1A). This loop is disordered in HsAGO2 crystal structures but adopts a defined conformation in AtAGO10. The AtAGO10 PIWI loop contacts the L2 domain adjacent to helix-7, which modulates the kinetics of seed pairing in HsAGO2 (Klum *et al*, 2017). PIWI-loop sequences are divergent between plant and animal AGOs (Fig EV1B). Thus, the PIWI loop was a likely candidate for dampening the plant AGO seed.

To test this idea, we created an AtAGO10 mutant (AtAGO10 + Hs-loop) in which the endogenous PIWI loop (residues 713–720) was replaced by the corresponding loop sequence from HsAGO2 (residues 602–608). The AtAGO10 + Hs-loop mutant bound a seed-matched target with a fivefold greater affinity than wild-type AtAGO10 (Fig 1C). Conversely, an HsAGO2 mutant with the PIWI loop from AtAGO10 (HsAGO2 + At-loop) bound the seed-matched target with an affinity matching that of AtAGO10 (Fig 1C). Most residues involved in stabilizing the PIWI-loop conformation are conserved in AtAGO1 (Fig EV2A) and clade 1 AGOs from evolutionary distant members of the plant kingdom (Fig EV1B). Swapping the PIWI-loop sequence of AtAGO1 into AtAGO10 had only modest effects on target affinity (Fig EV2B). Thus, miRNA-guided plant AGOs have stabilized PIWI loops that dampen seed pairing.

The PIWI loop increases the dynamic range of target recognition by AtAGO10

To understand how the PIWI loop affects miRNA-target pairing beyond the seed, we measured the affinities of wild-type AtAGO10 and AtAGO10 + Hs-loop for a series of target RNAs with increasing miRNA complementarity (Fig 2A; Table EV1). Target affinity of AtAGO10 varied 33-fold between targets with complementarity to g2–g8 ($K_D = 1.33 \pm 0.14$ nM) and g2–g21 ($K_D = 0.040 \pm 0.007$ nM; Fig 2B and C). Target affinities of AtAGO10 bearing the PIWI loop of AtAGO1 were similar (Fig EV2C and D). By contrast, AtAGO10 + Hs-loop target affinities varied only fourfold between targets with complementarity to g2–g8 (0.38 ± 0.03 nM) and g2–g21 (0.095 ± 0.012 nM; Fig 2B and C). The smaller affinity range is the result of increased affinity for the seed region and decreased contributions from downstream guide-target pairing. Thus, by weakening the strength of seed pairing, the structured PIWI loop effectively

 © 2023 The Authors

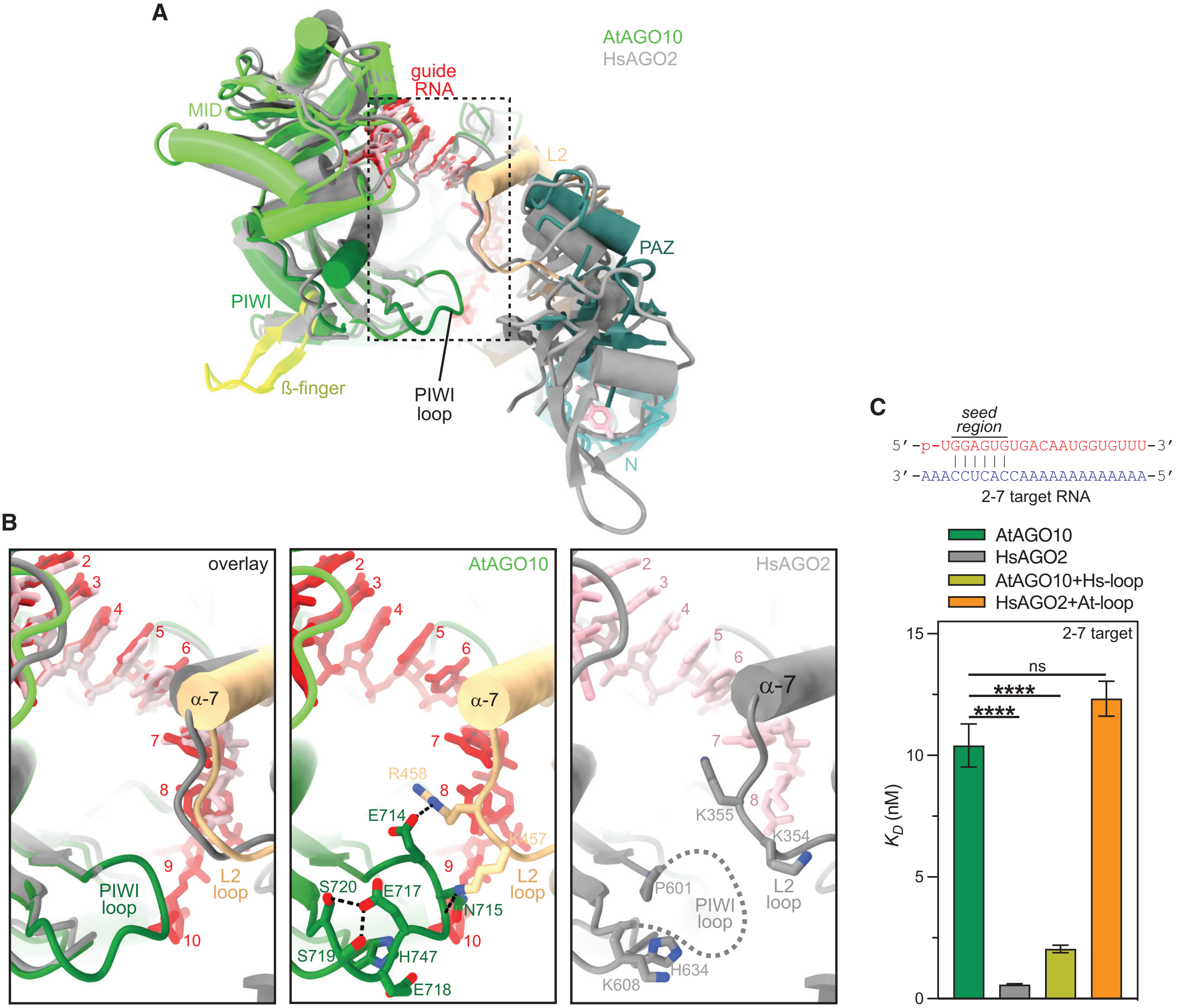

Figure 1. Structured PIWI domain loop distinguishes AtAGO10 from HsAGO2.

A Superposition of AtAGO10 (colored) and HsAGO2 (gray) bound to guide RNAs (red/pink).

B Close-up views of the boxed region in panel A showing the PIWI loop is ordered in AtAGO10 and disordered in HsAGO2.

C Dissociation constant values for wild-type AtAGO10, HsAGO2, and the AtAGO10 + Hs-loop HsAGO2 + At-loop mutants binding to a target RNA with base pairing complementarity to the miRNA seed region (guide nucleotides 2–7). Guide-target base pairing schematic is shown above. Plotted data represent the best-fit K_D values from $n = 3$ technical experimental trials, each containing 12 data points. Error bars indicate s.e.m. ANOVA with Dunnett's post hoc test P-values: ****< 0.0001. No data points were excluded from the analyses.

expands the dynamic range of miRNA-target recognition by AtAGO10.

The AtAGO10 PIWI loop connects stable seed pairing to downstream miRNA-target interactions

We also measured target release rates from wild-type AtAGO10 and AtAGO10 + Hs-loop (Fig 2D; Table EV1). The release of a target with complementarity to the extended seed region (2–8 target) from AtAGO10 followed a two-phase exponential, with ~85% of the target molecules released rapidly ($k_{off,fast} = 4.6\ min^{-1}$) and ~15% of the target molecules released 40-times more slowly ($k_{off,slow} = 0.12\ min^{-1}$) (Fig 2D). The observed biphasic kinetics indicate that wild-type AtAGO10 engages the 2–8 target in at least two distinct binding modes. By contrast, the release of the 2–8 target from AtAGO10 + Hs-loop was well described by a one-phase exponential, indicating a single binding mode. Notably, the rate of 2–8 target release from AtAGO10 + Hs-loop (0.13 min^{-1}) closely matches the $k_{off,slow}$ rate observed in the biphasic target release from wild-type AtAGO10 (Fig 2D). These results suggest that a minor fraction (~15%) of 2–8 target molecules fully engage the seed region when bound to wild-type AtAGO10 while the majority (~85%) of target

© 2023 The Authors

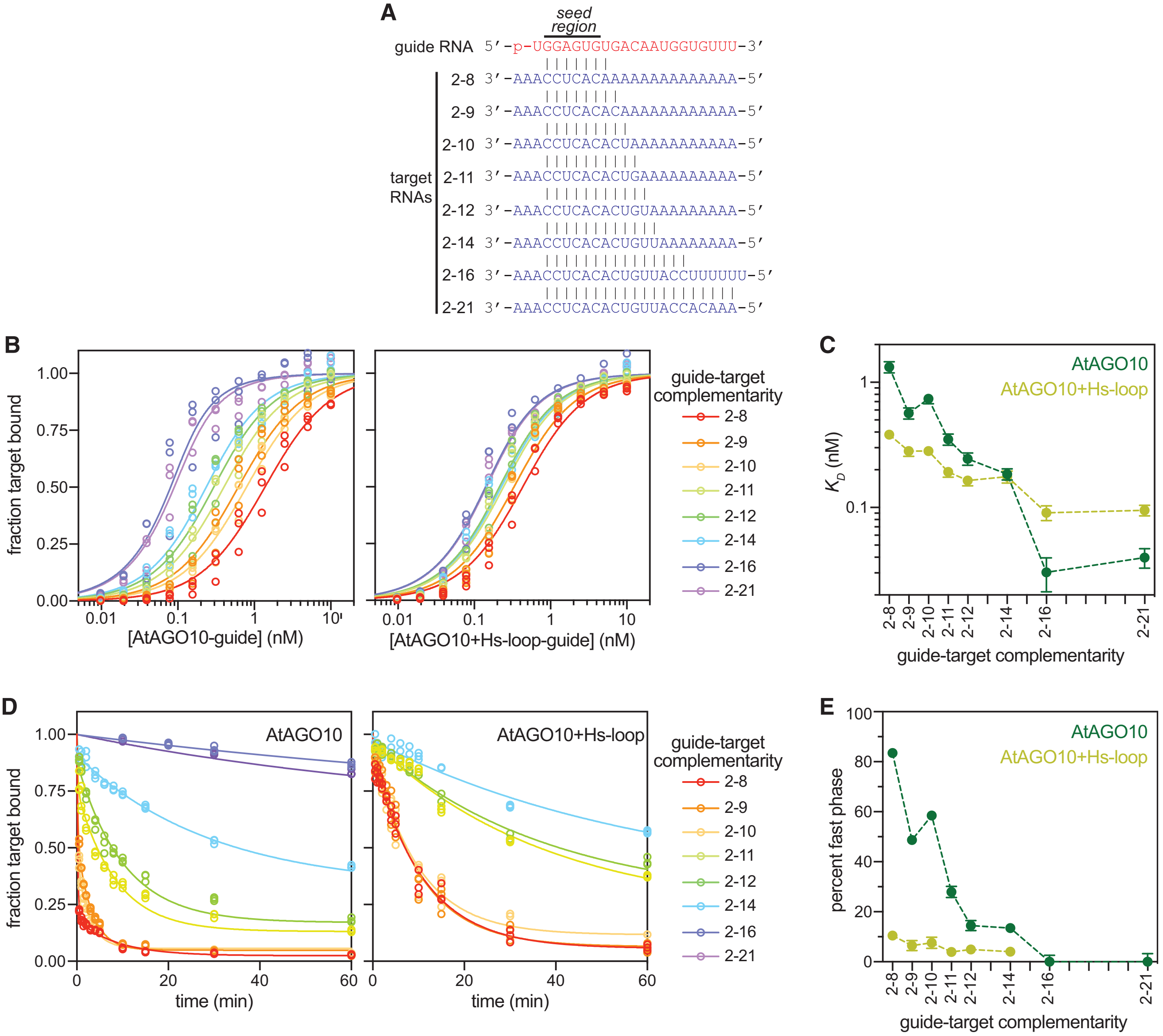

Figure 2. AtAGO10 PIWI loop is necessary for creating the extended MREs observed in plants.

A Base pairing schematic of guide RNA and target RNAs used in panels (B–E) and Fig 5.

B Fraction target RNA bound versus [AGO-guide] for wild-type AtAGO10 and the AtAGO10 + Hs-loop mutant.

C Dissociation constants derived from data in panel B.

D Fraction of ^{32}P-labeled target RNAs bound by AtAGO10 or AtAGO10 + Hs-loop after the addition of excess unlabeled target RNA versus time. Data were fit with a two-phase decay with a fixed $k_{off,fast}$ value for 4.6 min^{-1} for all curve fits.

E Fraction of target RNA released in the fast phase of curve fits in panel D.

Data information: Data points in (B and D) were measured in $n = 3$ independent technical trials. Data points in (C and E) represent best-fit K_D and percentage fast phase values to all data points in panels (B and D), respectively. Error bars indicate s.e.m. No data points were excluded from the analyses.

molecules do not. The fraction of target molecules released from AtAGO10 in the fast phase decreased with increasing guide-target complementarity (Fig 2E). Thus, for most binding events, the structured PIWI loop causes AtAGO10 to rapidly release seed-matched targets in the absence of downstream miRNA-target pairing.

Structural model for miRNA-targeting selectivity in plants

We propose that the difference in MRE size between plants and animals arises, to a large extent, from differences in plant and animal PIWI-loop structures. In this model, the stabilized AtAgo10 PIWI loop reaches across the central RNA-binding cleft to contact a loop

 © 2023 The Authors

in the L2 domain (Figs 3A and EV1A). This connection may dampen seed pairing by stabilizing the closed position of helix-7, which is directly tethered to the L2 loop and pivots between open and closed positions to facilitate the making and breaking of miRNA-target base pairs in the 3′ half of the seed (Schirle *et al*, 2014; Klum *et al*, 2017). By contrast, the PIWI loop in HsAgo2 is unstructured and thus cannot influence helix-7 (Figs 3B and EV1A). We expect miRNA-target pairing downstream of the seed physically disrupts the connection between PIWI and L2 loops, reducing the propensity of helix-7 to occupy its closed, seed-breaking position. The AtAgo10 PIWI loop thus makes stable seed pairing dependent on downstream miRNA-target interactions and thereby creates the extended MREs observed in plants (Allen *et al*, 2005).

The AtAGO10 PIWI loop adopts a structured conformation when swapped into HsAGO2

We next asked whether the AtAGO10 PIWI loop also adopts a stabilized conformation when swapped into HsAGO2. We determined the 2.5 Å resolution crystal structure of the HsAGO2 + At-loop mutant bound to the miRNA miR-122 (Fig 4A; Table EV2). The structure reveals that the swapped PIWI loop adopts a defined conformation (Fig 4B), likely stabilized by multiple internal hydrogen bonds within the loop: the sidechains of E602 and N603, the N603 mainchain carbonyl and E605 sidechain, and the sidechains of E605 and S608 are within hydrogen-bonding distances of each other. The stabilized loop reaches across the central cleft and forms two interactions with the L2 loop: The N603 sidechain is within hydrogen-bonding distance of the mainchain amide of K355, and the mainchain carbonyl of G604 may form a hydrogen bond with the sidechain of K354.

For direct comparison, we crystallized and determined the 2.5 Å resolution structure of wild-type HsAGO2 bound to miR-122 side-by-side with the mutant complex. In contrast to the HsAGO2 + At-loop mutant, there is no contiguous electron density corresponding to the center of the wild-type HsAGO2 PIWI loop (residues 603–606 are disordered), and no interactions between the PIWI loop and L2 loop are observed (Fig 4C). Except for the difference in the PIWI loops, the wild-type HsAGO2 and HsAGO2 + At-loop mutant structures are nearly identical, with an RMSD of 0.61 Å for all equivalent Cα atoms (Fig 4A). Thus, the swapped-in AtAGO10 PIWI loop adopts a defined conformation within HsAGO2 without otherwise impacting the overall structure.

AtAGO10 PIWI-loop insertion decreases the affinity of HsAGO2 for targets lacking extended complementarity

We compared the target affinities of the HsAGO2 + At-loop mutant to those of wild-type HsAGO2. Consistent with the hypothesis that a stabilized PIWI loop dampens seed pairing, HsAGO2 + At-loop bound the 2–8 target with ~75-fold lower affinity than wild-type HsAGO2 (Fig 5A and B; Table EV1). Moreover, of the target RNAs we examined, HsAGO2 + At-loop bound those with ≤ 15 base pairs of guide-target complementarity with an affinity more than an order lower than HsAGO2. By contrast, HsAGO2 + At-loop and wild-type HsAGO2 bound both 2–19 and 2–21 complementarity targets with comparable affinities (Fig 5A and B). Notably, target affinities of HsAGO2 + At-loop varied over 1,000-fold between the 2–8 target and the 2–21 target. This dynamic range is two orders of magnitude greater than the ninefold difference observed for the corresponding target affinities of wild-type HsAGO2. Thus, the HsAGO2 + At-loop mutant resembles *Drosophila* AGO2 (DmAGO2; Wee *et al*, 2012), an enzyme responsible for binding and destroying viral and transposon transcripts, in that HsAGO2 + At-loop binds far more tightly to fully complementary targets than to those matching only the seed.

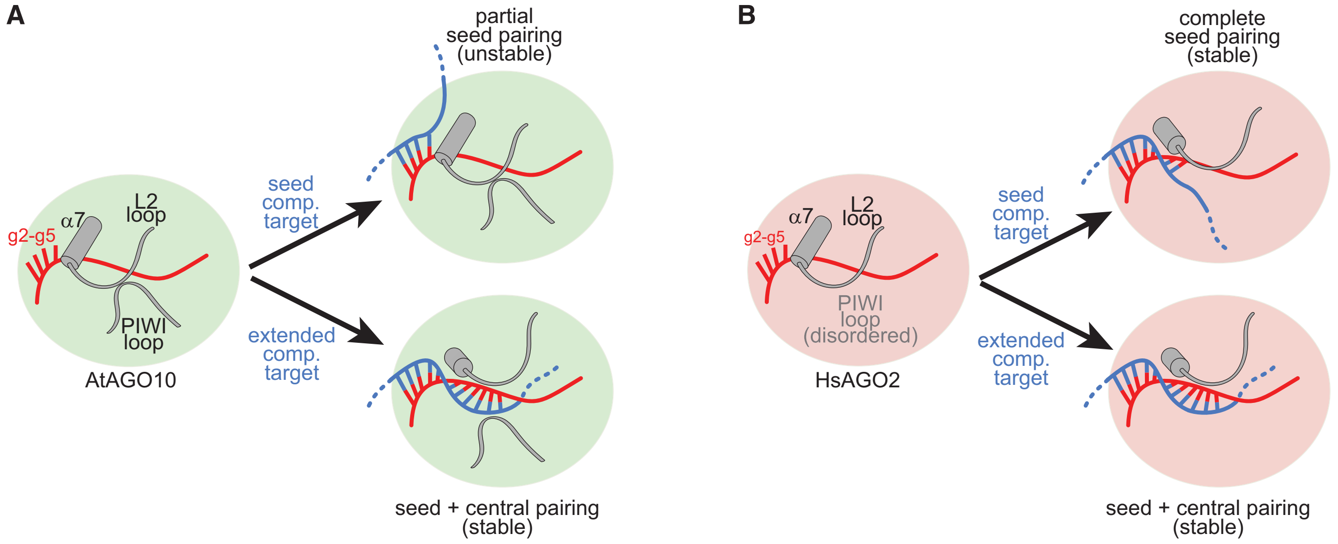

Figure 3. Proposed mechanism for seed dampening by the PIWI loop.

A, B Cartoon illustrating guide-target pairing beyond g5 is modulated by the position of helix-7 in AtAGO10 and HsAGO2. Interactions between the L2 and PIWI loops stabilize the closed, "seed-breaking," position of helix-7 in AtAGO10, but not in HsAGO2. Guide-target pairing beyond the seed disrupts interactions between the L2 and PIWI loops, stabilizing seed pairing.

© 2023 The Authors

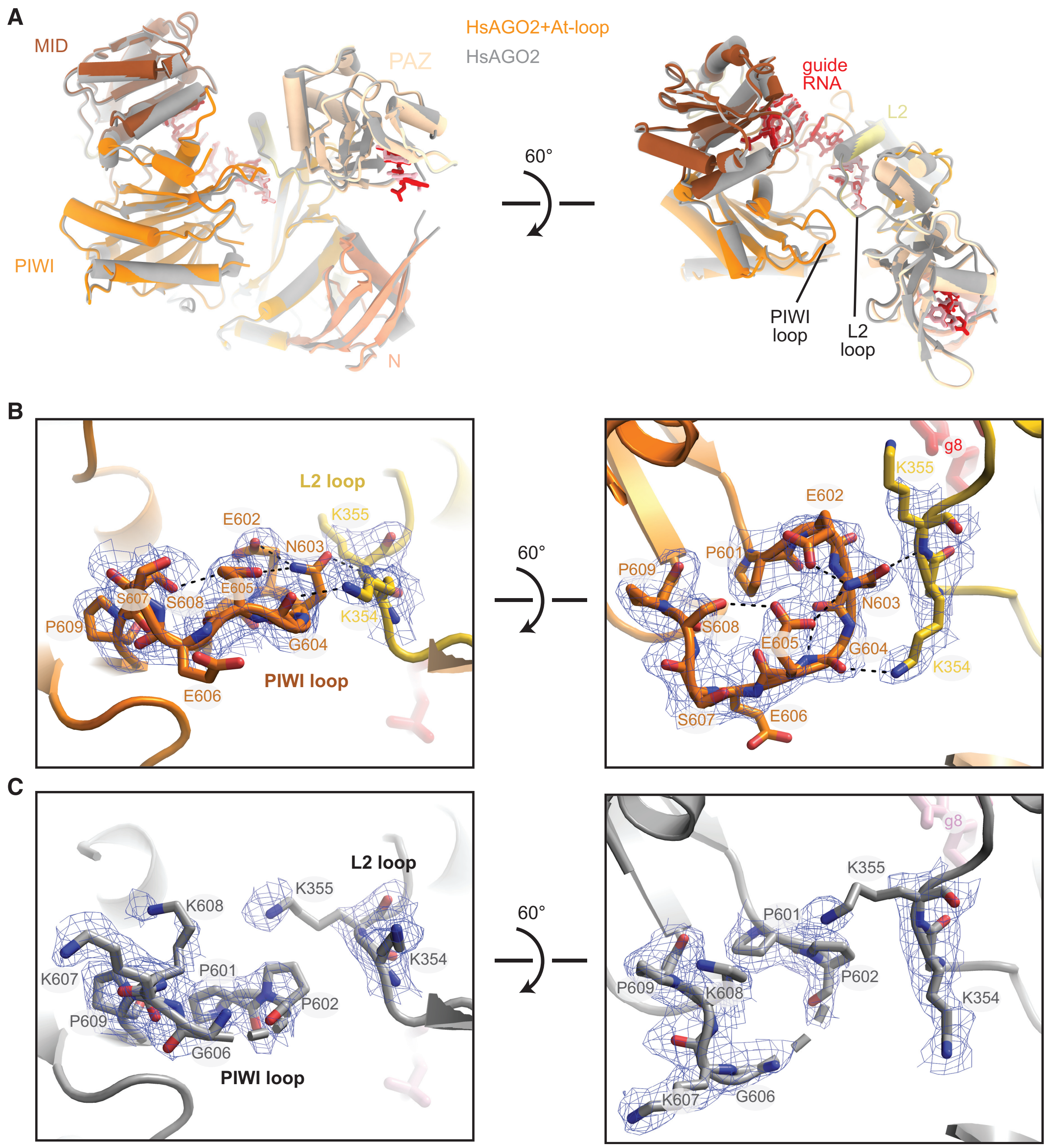

Figure 4. AtAGO10 PIWI loop adopts a stable conformation when swapped into HsAGO2.

A Superposition of wild-type HsAGO2 (gray) and the HsAGO2 + At-loop mutant (colored) crystal structures reveals nearly identical structures.

B Close-up view of PIWI loop in the HsAGO2 + At-loop mutant crystal structure. Blue mesh shows the final 2Fo-Fc electron density map.

C Close-up view of PIWI loop in the wild-type HsAGO2 crystal structure. Dashes indicate disordered residues in the PIWI loop. Blue mesh shows the final 2Fo-Fc electron density map.

Insertion of the AtAGO10 PIWI loop increases target cleavage turnover by HsAGO2

We next compared rates of target RNA cleavage by wild-type HsAGO2 and the HsAGO2 + At-loop mutant. When incubated in excess of target RNAs, wild-type HsAGO2 and HsAGO2 + At-loop converted target substrates into cleaved products at similar rates (Fig EV3A). By contrast, when AGO2 was limiting, the cleavage reaction catalyzed by HsAGO2 + At-loop was faster than wild-type HsAGO2 (Fig EV3B). Taken with previous results showing product release is often the rate-limiting step in AGO-catalyzed RNA cleavage (Haley & Zamore, 2004; Rivas *et al*, 2005; Wee *et al*, 2012;

 © 2023 The Authors

Deerberg *et al*, 2013; Salomon *et al*, 2015), this observation suggested that the AtAGO10 PIWI loop accelerates the release of cleaved products. To test this idea, we monitored the cleavage of a 2–19 complementary target by HsAGO2 and HsAGO2 + At-loop samples over short time periods, before appreciable substrate depletion and production inhibition (Fig 5C and D). Fitting the time course data to the burst-and-steady-state equation (Arif *et al*, 2022) shows that product release (k_3) is 3.9 ± 1.8 times faster for HsAGO2 + At-loop than wild-type HsAGO2 at 36°C (Fig 5D and E; Table EV1). Cleavage of bound substrates (k_2) may be slower in the HsAGO2 + At-loop mutant, but the differences between wild-type and At-loop mutant k_2 values are not significant in our data. The combined results show that swapping the AtAGO10 PIWI loop into HsAGO2 creates an HsAGO2 variant with decreased affinity for targets lacking sufficient complementarity to be cleaved and accelerated the turnover of targets that are cleaved.

Swapping the AtAGO10 PIWI loop into HsAGO2 improves siRNA-mediated silencing in mammalian cells

After finding that HsAGO2 + At-loop has plant AGO-like biochemical activity *in vitro*, we wondered whether the engineered HsAGO2 has enhanced siRNA-mediated silencing properties in mammalian cells. We transfected various amounts of the purified HsAGO2 + At-loop-guide RNA complex into HEK293 cells along with luciferase reporters bearing complementary sites to either nucleotides 2–8 (miRNA-target) or 2–19 (siRNA-target) of the guide RNA (Fig 6A). For comparison, we also transfected HEK293 cells with the luciferase reporters and either the wild-type HsAGO2-guide RNA complex or an equivalent amount of siRNA duplex.

The engineered HsAGO2 + At-loop-guide RNA complex repressed the miRNA-target an average of 1.1-fold relative to transfection of the reporter alone (Fig 6B). This difference was not significant in

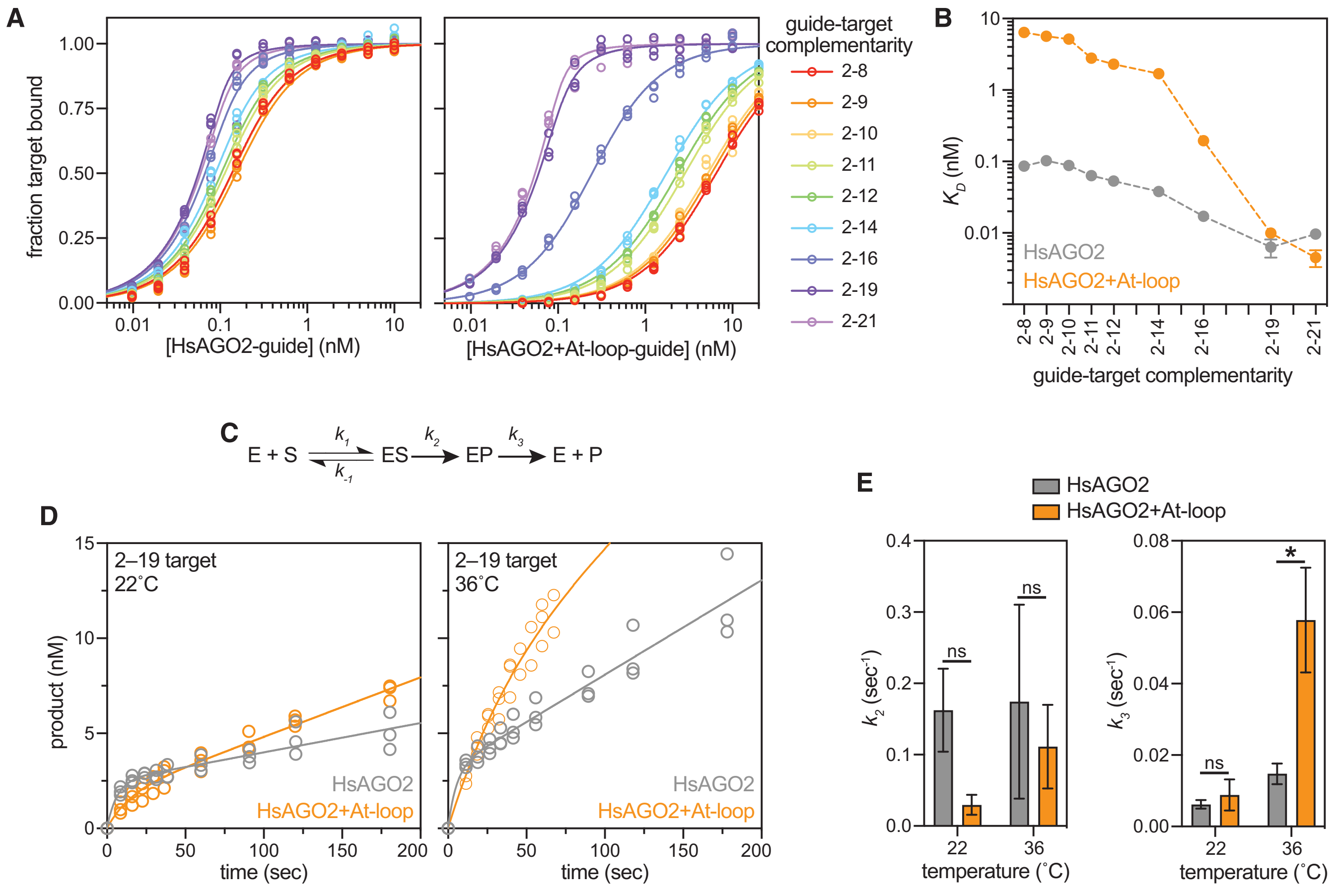

Figure 5. Swapping the AtAGO10 PIWI loop into HsAGO2 increases targeting selectivity and catalytic turnover.

A Fraction target RNA bound versus [AGO-guide] for wild-type HsAGO2 and the HsAGO2 + At-loop mutant. Guide and target RNAs used are the same as shown in Fig 2A.

B Dissociation constants derived from data in panel A.

C Target cleavage reaction scheme. E indicates enzyme, S is the target RNA substrate, and P is the cleaved product.

D Concentration of cleaved product generated by HsAGO2 or HsAGO2 + At-loop at 22°C or 36°C versus time.

E k_2 and k_3 values derived from fitting data in D to the burst-and-steady-state equation (see Materials and Methods).

Data information: Data points in panels (A and D) were obtained in $n = 3$ independent technical trials. Data in panels (B and E) represent best-fit values of data in panels (A and D), respectively. Error bars indicate standard error. Unpaired t-test P-value: *0.0447. No data points were excluded from the analyses.

© 2023 The Authors

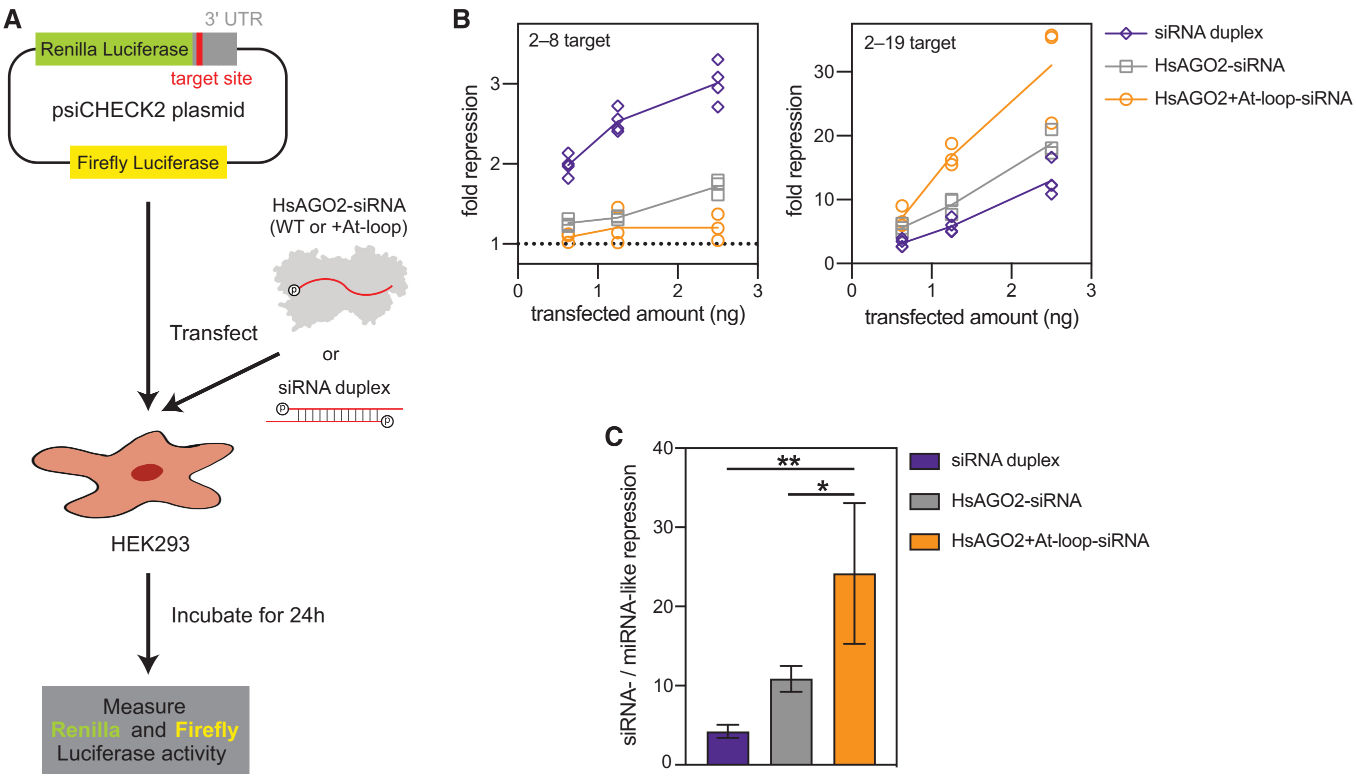

Figure 6. HsAGO2 + At-loop has improved RNAi potency in mammalian cells.

A Schematic of the RNAi experiment. HEK293 cells were transfected with a plasmid encoding *Renilla* luciferase with a single target site and targeting wild-type or + At-loop mutant HsAGO2-siRNA complexes, or siRNA duplexes.

B Fold repression of *Renilla* expression (compared with reporter plasmid transfection alone) versus the amount of transfected siRNA duplex, wild-type HsAGO2-siRNA complex, or HsAGO2 + At-loop-siRNA complex. The 2–8 target (left panel) indicates miRNA-like targeting. The 2–19 target (right panel) indicates siRNA-like targeting. Dotted line indicates the normalized expression level of the positive control (no repression).

C Ratio of the average level of siRNA-like fold repression to the average level of miRNA-like fold repression at the highest transfected amounts in panel B.

Data information: Graphed data in (B and C) represent the individual and the ratio of mean values of $n = 3$ independent biological trials, respectively. Error bars indicate s.e.m. ANOVA with Dunnett's post hoc test *P*-values: **0.0062, *0.0363. No data points were excluded from the analyses.

our data. Wild-type HsAGO2-guide RNA and the siRNA duplex both displayed stronger miRNA-like targeting, repressing the miRNA-target 1.3–1.7-fold and 2.1–3.3-fold, respectively. By contrast, HsAGO2 + At-loop effectively silenced the siRNA-target, reducing expression 7–31-fold (Fig 6B). Wild-type HsAGO2-guide RNA and the siRNA duplex were both less potent, repressing the siRNA-target 5–19-fold and 3–13-fold, respectively (Fig 6B). Thus, consistent with *in vitro* measurements, the engineered HsAGO2 + At-loop displays superior RNAi properties, with enhanced siRNA-like targeting silencing and reduced miRNA-like targeting in mammalian cells (Fig 6C).

Discussion

Argonaute proteins have long been known to reshape the fundamental properties of RNA:RNA hybridization for guide-target pairing (Wee *et al*, 2012). This feature enables AGOs to conduct rapid and accurate target searches (Chandradoss *et al*, 2015; Salomon *et al*, 2015). Based on our results and the previous work of others (Wee *et al*, 2012; Iwakawa & Tomari, 2013; Becker *et al*, 2019; Ober-Reynolds *et al*, 2022), we suggest that controlling guide-target hybridization also allows different AGOs to adapt distinct small RNA classes to discrete biological roles. We show that the identity of a small PIWI domain loop can have dramatic effects on guide-target hybridization in the miRNA seed, contributing to the distinct MREs found in plants and animals. Notably, the PIWI loop does not directly contact the guide or target nucleotides involved in seed pairing (Fig 1). We, therefore, suggest that the PIWI loop shapes guide-target hybridization by contributing to conformational dynamics of the AGO-guide complex. These dynamics likely include the movements of helix-7, which influences rates of making and breaking seed pairing (Schirle *et al*, 2014; Klum *et al*, 2017). Supporting this model, swapping the PIWI loop of AtAGO1 into AtAGO10, which we predict removes one contact modulating helix-7 (due to histidine in the place of E741 of AtAGO10) led to a small but significant increase in affinity for a seed-matched target RNA (Fig EV2A and B). It is worth noting that the PIWI-loop sequences are generally conserved within each plant AGO clade but not between clades (Fig EV1B). We hypothesize that these variations may connect to the diversification of AGO function in plants.

Our results also reveal that additional small structural features, beyond the PIWI loop, must further tune guide-target hybridization. Although swapping PIWI loops made AtAGO10 more animal-like

© 2023 The Authors

and HsAGO2 more plant-like with respect to seed pairing (Fig 1C), neither PIWI-loop mutant perfectly mimicked its trans-kingdom counterpart with respect to recognizing a broad range of target types (Fig EV3C). The identities of the structural elements that further tune AGO dynamics and guide-target hybridization remain to be discovered, but likely candidates include: (i) the L2 loop (that contacts the PIWI loop, see Figs 1A and EV1A), which adopts distinct conformations in AtAGO10 and HsAGO2, and forms a "central-gate" that modulates pairing to the central region of guides bound to HsAGO2 (Sheu-Gruttadauria *et al*, 2019b; Anzelon *et al*, 2021); (ii) the "eukaryotic insertion," which is adjacent to the PIWI loop and has been shown to impact HsAGO2 target affinity in a phosphorylation-dependent manner (Golden *et al*, 2017; Quevillon Huberdeau *et al*, 2017; Bibel *et al*, 2022); and (iii) the stalk of the L1 domain, which creates the primary hinge by which both AGO and PIWI proteins bend in response to guide-target pairing (Schirle *et al*, 2014; Sheu-Gruttadauria *et al*, 2019a, 2019b; Anzelon *et al*, 2021).

Finally, although RNAi is usually induced via the introduction of siRNA duplexes into target cells or tissues, the direct introduction of preassembled HsAGO2-siRNA complexes can also induce the knockdown target mRNAs (Castellanos-Gonzalez *et al*, 2016; Li *et al*, 2018). This approach is particularly useful in organisms that lack RNAi components (Castellanos-Gonzalez *et al*, 2016), or when a delivery payload with chemical properties distinct from naked RNA is desired (Li *et al*, 2018). Our results indicate that this approach could be further advanced through the development of engineered HsAGO-siRNA complexes. We suggest that by incorporating the AtAGO10 PIWI loop, and perhaps other small changes (i.e., point mutations that disrupt interactions with miRNA-associated silencing factors (Sheu-Gruttadauria & MacRae, 2018)), into HsAGO2 it may be possible to create an AGO-guide complex that is nonimmunogenic and optimized for RNAi in humans.

Materials and Methods

Bacterial strains and plasmids

Bacteria used for cloning were chemically competent *E. coli* OmniMAX™ (C854003, Thermo Fisher). Bacteria used for the production of bacmid DNAs were DH10Bac™ chemically competent *E. coli* (10361012, Thermo Fisher).

Bacterial media and growth conditions

All bacterial cultures were grown in Luria–Bertani (LB) medium at 37°C. When needed, media was supplemented with one or more of the following antibiotics at the following concentrations: ampicillin (100 μg/ml), kanamycin (40 μg/ml), tetracycline (5 μg/ml), gentamycin (7 μg/ml), 5-Bromo-4-Chloro-3-Indolyl β-D-Galactopyranoside (X-gal, 25 μg/ml in dimethylformamide), and/or Isopropyl β-D-1-thiogalactopyranoside (IPTG, 1 mM).

Insect cell media and growth conditions

Sf9 cells (94-001S, Expression Systems) were grown in Lonza Insect XPRESS™ medium supplemented with 1× Gibco™ Antibiotic-Antimycotic in suspension at 27°C.

Cloning and mutagenesis

The plasmid encoding AtAGO10 + Hs-loop was produced by NEBuilder® HiFi DNA Assembly. First, 5′ and 3′ fragments encoding the AtAGO10 + Hs-loop coding sequence were generated by PCR using the wild-type AtAGO10 coding sequence as a template and DNA primers with complementary to AtAGO10 and containing the Hs-loop sequence (Table EV3). PCR products were purified on an agarose gel and assembled into pFastBac HTA (digested by SalI) using the NEBuilder® HiFi DNA Assembly Master Mix. The plasmid encoding HsAGO2 + At-loop was produced in a manner similar to the AtAGO10 mutant except the HsAGO2 coding sequence was used as the PCR template with HsAGO2-specific primers (see Table EV3).

Preparation of AtAgo10-guide RNA complex

His_6-Flag-Tev-tagged AtAgo10 proteins were expressed in Sf9 cells using a baculovirus system (Invitrogen) as described previously (Xiao & MacRae, 2022). 60 h cultured 750 ml 3.4×10^6 cells/ml Sf9 cells infected with ~15 ml virus were harvested by centrifugation. Usually, two of these cell pellets were combined and suspended in ~200 ml Lysis Buffer (50 mM NaH2PO4, pH 8, 300 mM NaCl, 5% glycerol, 20 mM imidazole, 0.5 mM TCEP). Resuspended cells were lysed by passing twice pass through a M-110P lab homogenizer (Microfluidics). The resulting total cell lysate was clarified by centrifugation (30,000 *g* for 25 min), and the soluble fraction was applied to 8 ml packed Ni-NTA resin (Qiagen) and gently rocked at 4°C for 1.5 h in 50 ml conical tubes. Resin was pelleted by brief centrifugation, and the supernatant solution was discarded. The resin was washed with ~50 ml ice-cold Nickel Wash Buffer (300 mM NaCl, 20 mM imidazole, 0.5 mM TCEP, 50 mM Tris, pH 8). Centrifugation/wash steps were repeated a total of three times. Co-purified cellular RNAs were degraded by incubating with 400 U micrococcal nuclease (Clontech) on-resin in ~25 ml of Nickel Wash Buffer supplemented with 5 mM CaCl2 at room temperature for ~1 h. The nuclease-treated resin was washed three times again with Nickel Wash Buffer and then eluted in four column volumes of Nickel Elution Buffer (300 mM NaCl, 300 mM imidazole, 0.5 mM TCEP, 50 mM Tris, pH 8). Eluted protein was incubated with 24 nmol synthetic guide RNA and 150 μg TEV protease during ~2 h dialysis against 1 L of Dialysis Buffer (300 mM NaCl, 0.5 mM TCEP, 50 mM Tris, pH 8) at 4°C. After dialysis, the capture resin was prepared by incubating 345.6 uL packed High Capacity Neutravidin Resin (Thermo Fisher) with 28.8 nmol capture oligo in wash A buffer (100 mM KOAc, 2 mM MgOAc, 0.01% CHAPS, 30 mM Tris, pH 8) for 30 min at 4°C, following by 10 ml wash A buffer wash. Dialyzed protein supplemented with 0.01% CHAPS and 2 mM MgOAc was then incubated with capture resin at RT for 1 h (without rocking!!!! Just gently inverting the tube every 5–10 min). Then, the resin was washed by three times with 10 ml Wash A followed by three times with 10 ml Wash B (2 M KOAc, 2 mM MgOAc, 0.01% CHAPS, 30 mM Tris, pH 8) and Wash C (1 M KOAc, 2 mM MgOAc, 0.01% CHAPS, 30 mM Tris, pH 8). The resin was then resuspended in 1,900 μl Wash C with 57.6 nmol competitor DNA at RT for ~2 h (gently inverting the tube every 5-10 min). The elute was dialyzed in 1 L Q dialyzing buffer (150 mM NaCl, 0.01% CHAPS, 0.5 mM TCEP, 20 mM Tris, pH 8) at 4°C overnight. After dialysis, 240 μl Q Sepharose Fast Flow anion exchange resin slurry (GE Healthcare) was equilibrated in Q dialyzing buffer.

© 2023 The Authors

The dialyzed protein was then passed through this resin to remove unbound oligonucleotides, and flow-through solution was collected. The flow-through was then concentrated while buffer exchanging to 1–3 mg/ml in Tris Crystal buffer (10 mM Tris pH 8, 100 mM NaCl, 0.5 mM TCEP). The concentrated protein was aliquoted, flash-frozen in liquid N_2, and stored at −80°C. The concentration of the AtAgo10-guide RNA complex was determined by Bradford assay with BSA as a standard.

Preparation of HsAgo2-guide RNA complex

His_6-Flag-Tev-tagged HsAgo2 proteins were expressed in Sf9 cells using a baculovirus system (Invitrogen) as described previously (Sheu-Gruttadauria & MacRae, 2018). 60 h cultured 750 ml 3.4×10^6 cells/ml Sf9 cells infected with ~ 15 ml virus were harvested by centrifugation. One pellet was suspended in ~ 100 ml Lysis Buffer (50 mM NaH2PO4, pH 8, 300 mM NaCl, 5% glycerol, 20 mM imidazole, 0.5 mM TCEP). Resuspended cells were lysed by passing twice pass through a M-110P lab homogenizer (Microfluidics). The resulting total cell lysate was clarified by centrifugation (30,000 *g* for 25 min), and the soluble fraction was applied to 3 ml packed Ni-NTA resin (Qiagen) and gently rocked at 4°C for 1.5 h in 50 ml conical tubes. Resin was pelleted by brief centrifugation, and the supernatant solution was discarded. The resin was washed with ~50 ml ice-cold Nickel Wash Buffer (300 mM NaCl, 20 mM imidazole, 0.5 mM TCEP, 50 mM Tris, pH 8). Centrifugation/wash steps were repeated a total of three times. Co-purified cellular RNAs were degraded by incubating with 200 U micrococcal nuclease (Clontech) on-resin in ~ 25 ml of Nickel Wash Buffer supplemented with 5 mM CaCl2 at room temperature for ~1 h. The nuclease-treated resin was washed three times again with Nickel Wash Buffer and then eluted in four column volumes of Nickel Elution Buffer (300 mM NaCl, 300 mM imidazole, 0.5 mM TCEP, 50 mM Tris, pH 8). Eluted protein was incubated with 24 nmol synthetic guide RNA and 150 µg TEV protease during an overnight dialysis against 1–2 l of Dialysis Buffer (300 mM NaCl, 0.5 mM TCEP, 50 mM Tris, pH 8) at 4°C. After dialysis, the capture resin was prepared by incubating 345.6 µl packed High Capacity Neutravidin Resin (Thermo Fisher) with 28.8 nmol capture oligo in wash A buffer (100 mM KOAc, 2 mM MgOAc, 0.01% CHAPS, 30 mM Tris, pH 8) for 30 min at 4°C, following by 10 ml wash A buffer wash. Dialyzed protein supplemented with 0.01% CHAPS and 2 mM MgOAc was then incubated with capture resin at RT for 1 h (without rocking!!!! Just gently inverting the tube every 5–10 min). Then, the resin was washed by three times with 10 ml Wash A followed by three times with 10 ml Wash B (2 M KOAc, 2 mM MgOAc, 0.01% CHAPS, 30 mM Tris, pH 8) and Wash C (1 M KOAc, 2 mM MgOAc, 0.01% CHAPS, 30 mM Tris, pH 8). The resin was then resuspended in 1,900 µl Wash C with 57.6 nmol competitor DNA at RT for ~ 2 h (gently inverting the tube every 5-10 min). The elute was dialyzed in 1 l Q dialyzing buffer (150 mM NaCl, 0.01% CHAPS, 0.5 mM TCEP, 20 mM Tris, pH 8) at 4°C for 1 h and then moved to fresh 1 l Q dialyzing buffer for another 2 h. When the dialyzing was near completion, 240 µl Q Sepharose Fast Flow anion exchange resin slurry (GE Healthcare) was equilibrated in Q dialyzing buffer. The dialyzed protein was then passed through this resin to remove unbound oligonucleotides, and the flow-through solution was collected. The flow-through was then concentrated while buffer exchanging to 1–3 mg/ml in Tris Crystal buffer (10 mM Tris pH 8, 100 mM NaCl, 0.5 mM TCEP). The concentrated protein was aliquoted, flash-frozen in liquid N_2, and stored at −80°C. The concentration of the HsAgo2-guide RNA complex was determined by Bradford assay with BSA as a standard.

Equilibrium target binding assays

Equilibrium dissociation constants were determined as described previously (Schirle *et al*, 2014). Briefly, various concentrations of the AtAgo10_D795A-guide RNA, AtAgo10 + Hs-loop_D795A-guide RNA, HsAGO2-guide RNA or HsAGO2 + At-loop-guide RNA samples were incubated with 0.1 nM ^{32}P 5′-radiolabeled target RNA in binding reaction buffer (30 mM Tris pH 8, 100 mM potassium acetate, 2 mM Magnesium acetate, 0.5 mM TCEP, 0.005% (v/v) NP-40, 0.01 mg/ml baker's yeast tRNA), in a reaction volume of 100 µl at room temperature for 60 min. Using a dot-blot apparatus (GE Healthcare Life Sciences), protein-RNA complexes were captured on Protran nitrocellulose membrane (0.45 µm pore size, Whatman, GE Healthcare Life Sciences) and unbound RNA on Hybond Nylon membrane (Amersham, GE Healthcare Life Sciences). A vacuum was applied to pull samples through the membranes, and wells were immediately washed once with 100 µl of ice-cold wash buffer (30 mM Tris pH 8.0, 100 mM potassium acetate, 2 mM Magnesium acetate, 0.5 mM TCEP). Membranes were air-dried, and the ^{32}P signal was visualized by phosphor imaging. ImageQuant (GE Healthcare Life Sciences) was used to quantify data and dissociation constants calculated using Prism version 6.0 g (GraphPad Software, Inc.), using the following formula, which accounts for potential ligand depletion (Wee *et al*, 2012):

$$F = B_{max}\frac{([E_T] + [S_T] + K_D) - \sqrt{([E_T] + [S_T] + K_D)^2 - 4[E_T][S_T]}}{2[S_T]}$$

where F = fraction of target bound, B_{max} = calculated maximum number of binding sites, $[E_T]$ = total enzyme concentration, $[S_T]$ = total target concentration, and K_D = apparent equilibrium dissociation constant.

Target dissociation assay

Target dissociation rates were determined by incubating AtAgo10_D795A-guide RNA, AtAgo10 + Hs-loop_D795A-guide RNA, HsAGO2-guide RNA, or HsAGO2 + At-loop-guide RNA samples with 0.1 nM ^{32}P 5′-radiolabeled target RNA in binding reaction buffer (30 mM Tris pH 8.0, 100 mM potassium acetate, 2 mM Magnesium acetate, 0.5 mM TCEP, 0.005% (v/v) NP-40, 0.01 mg/ml baker's yeast tRNA) in a single reaction with a volume of 100 µl per time point planned for the experiment (e.g., 1,000 µl for 10 time points) at room temperature for 60 min. The concentration of protein complex was 5 nM for 2–8 target or 2.5 nM for all other targets measured. After sample equilibration, a zero-time point was taken by applying 100 µl of the reaction to the dot-blot apparatus under vacuum, followed by 100 µl of ice-cold wash buffer (30 mM Tris pH 8.0, 100 mM potassium acetate, 2 mM Magnesium acetate, 0.5 mM TCEP). The dissociation time course was started by the addition of 300 nM (final concentration) unlabeled target RNA. Aliquots of 100 µl were taken at various times and immediately applied to a dot-blot

 © 2023 The Authors

apparatus under vacuum, followed by 100 µl of ice-cold wash buffer. Time points ranged from 0.25 to 100 min. Membranes were air-dried and visualized by phosphorimaging. Quantification of the 32P signal was performed using ImageQuant TL (GE Healthcare). The fraction of target RNA bound was calculated as the ratio of bound to total (bound + free) target RNA for various concentrations of AGO-guide complexes. Dissociation rates were calculated by plotting data as fraction bound versus time and fitting to a two-phase decay curve with shared k_{fast} value using Prism v.8.0 (GraphPad).

Target cleavage assay

Initial slicing assays

For single turnover slicing assays, purified HsAGO2-guide RNA or HsAGO2 + At-loop-guide RNA complex (10 nM, final concentration) was incubated at 37°C with complementary ^{32}P 5′-radiolabeled target RNAs (2 nM, final concentrations) in reaction buffer composed of 30 mM Tris pH 8.0, 100 mM potassium acetate, 2 mM Magnesium acetate, 0.5 mM TCEP, and 0.01 mg/ml baker's yeast tRNA (Fig EV3). For multiple turnover slicing, purified HsAGO2-guide RNA complexes or HsAGO2 + At-loop-guide RNA complex (1 nM, final concentration) was incubated at 37°C with complementary ^{32}P 5′-radiolabeled target RNAs (5 nM, final concentrations) in reaction buffer. Target cleavage was stopped at various times by mixing aliquots of each reaction with an equal volume of denaturing gel loading buffer (98% w/v formamide, 0.025% xylene cyanol, 0.025% w/v bromophenol blue, 10 mM EDTA pH 8.0). Intact and cleaved target RNAs were resolved by denaturing PAGE (15%) and visualized by phosphorimaging. Quantification of signal was performed using ImageQuant TL (GE Healthcare).

Kinetic constant rate determination

The concentrations of active enzyme in our HsAGO2 and HsAGO2 + t-loop preparations were first determined by fitting preliminary room temperature (22°C) slicing time course data to the burst-and-steady-state equation for a diffusion-limited enzyme (Arif *et al*, 2022; Fig 5D):

$$[\mathrm{P}] = f(t) = E_0 \times \left(\left(\frac{k_2}{(k_2 + k_3)} \right)^2 \times \left(1 - e^{-t(k_2+k_3)} \right) + t \times k_2 \times \frac{k_3}{(k_2 + k_3)} \right)$$

where [P] is the concentration of cleaved product at time t, E_0 is the concentration of active enzyme, k_2 is the first order rate constant describing the cleavage of target RNAs bound to the enzyme, and k_3 is the first order rate constant describing release of cleaved targets from the enzyme (see Fig 5C). The temperature of 22°C was used for the initial determination of active enzyme concentration in our AGO preparations because the 22°C reactions proceed more slowly than 37°C and display greater differences between k_2 and k_3, making E_0 determination more reliable. The known concentrations of active enzyme were then used to set up three independent slicing time course trials with $[E_0] = 5$ nM and [target RNA] = 100 nM at both 22°C and 37°C. Data points were taken rapidly enough to ensure that sufficient information for curve fitting was obtained before > 15% of target RNA was consumed. Cleaved and uncleaved target RNAs were visualized and quantified as described above. Data were fit to the burst-and-steady-state equation using Prism v.8.0 (GraphPad).

Crystallization and diffraction data collection

HsAGO2-miR122 and HsAGO2 + At-loop-miR122 crystals were grown by hanging drop vapor diffusion at 20°C and appeared in 24 h. Drops contained a 1.0:0.8 ratio of protein (1 mg/ml) to reservoir solution (0.1 M Tris pH8, 10 mM MgCl2, 0.1 M Phenol, 15% PEG3350, 10% Isopropanol). After growing for 1 week, crystals were harvested for x-ray data collection by soaking first in a reservoir solution containing 25% ethylene glycol as cryo-protectant. Following cryo-protection, crystals were cryo-cooled by plunging into liquid N2. Data were collected on beamlines 9–2 at the Stanford Synchrotron Radiation Lightsource (SSRL). Data were processed using XDS and Aimless (Kabsch, 2010; Winn *et al*, 2011).

Model building and refinement

The HsAGO2-miR122 and HsAGO2 + At-loop-miR122 structures were solved by molecular replacement using a previously determined guide-only structure (PDB:4OLA) as search models with Phaser-MR in the PHENIX graphical user interface (Adams *et al*, 2010). Models were built using Coot (Emsley *et al*, 2010) and were submitted to XYZ coordinate, Occupancies, and B-factor refinement using PHENIX. Model building and refinement continued iteratively until all interpretable electron density was modeled. Water molecules were updated automatically in PHENIX. All structure figures were generated with PyMOL (Schrödinger, LLC).

Luciferase assay

Target sites were cloned into psiCHECK2 plasmid (Promega Corporation) using Xho1 and Not1 sites. The siRNA duplex was made by denaturing 1:1 mixture of guide and passenger strands in annealing buffer (10 mM Tris, pH8.0, 50 mM NaCl) at 95°C for 2 min and then cooling to room temperature by placing on the benchtop. Luciferase reporter assays were performed with human HEK293 cells (CRL-1573, ATCC). The HEK293 cells were plated at 10^4 cells with 80ul DMEM complete media per well in a 96-well white plate (Thermal Scientific, Cat#136101) and cultured in a temperature-, CO^{2}-, and humidity-controlled cell incubator for ~4 h to allow cells to attach to the bottom of the plate. Cells in each well were first transfected with 40 ng psiCHECK2 plasmid using Lipofectamine 2000 (Invitrogen) based on the manufacturer's protocol. Then, 2.5 µl 2.5 pmol siRNA duplex, HsAGO2-siRNA or HsAGO2 + At-loop-siRNA complexes was made in Optimem (Invitrogen) and mixed with 0.4 µl Lipofectamine 2000 in 2.1 µl Optimem for a total volume of 5 µl per well. The volume of the master mix made is based on how many wells are needed. 1.25 pmol transfection mix is made by diluting 2.5 pmol transfection mix in an equal value of Optimem, and 0.63 pmol transfection mix is made by diluting 1.25 pmol transfection mix in an equal value of Optimem. Each transfection condition was prepared at least in triplicate. After a 24-h incubation, media in each well were removed, and luciferase activities were measured using Dual-Glo® Luciferase Assay System (Promega Corporation, Cat #E2920) based on the manufacturer's description.

Blinding was not deliberately used in any experiments.

© 2023 The Authors

Data availability

Atomic coordinates and structure factors for wild-type HsAGO2 (8D71; http://identifiers.org/pdb/8D71) and HsAGO2-At-loop (8D6J; http://identifiers.org/pdb/8D6J), both bound to miR-122, have been deposited in the PDB.

Expanded View for this article is available online.

Acknowledgments
We are grateful to MacRae lab members for their advice. The research was funded by NIH grant R35GM127090 to I.J.M.

Author contributions
Yao Xiao: Conceptualization; formal analysis; investigation; visualization; methodology; writing – original draft. **TingYu M Liu:** Validation; investigation. **Ian J MacRae:** Conceptualization; formal analysis; supervision; funding acquisition; validation; investigation; writing – review and editing.

Disclosure and competing interests statement
The authors declare that they have no conflict of interest.

References

Adams PD, Afonine PV, Bunkoczi G, Chen VB, Davis IW, Echols N, Headd JJ, Hung LW, Kapral GJ, Grosse-Kunstleve RW *et al* (2010) PHENIX: a comprehensive python-based system for macromolecular structure solution. *Acta Crystallogr D Biol Crystallogr* 66: 213–221

Allen E, Xie Z, Gustafson AM, Carrington JC (2005) microRNA-directed phasing during trans-acting siRNA biogenesis in plants. *Cell* 121: 207–221

Anzelon TA, Chowdhury S, Hughes SM, Xiao Y, Lander GC, MacRae IJ (2021) Structural basis for piRNA targeting. *Nature* 597: 285–289

Arif A, Bailey S, Izumi N, Anzelon TA, Ozata DM, Andersson C, Gainetdinov I, MacRae IJ, Tomari Y, Zamore PD (2022) GTSF1 accelerates target RNA cleavage by PIWI-clade Argonaute proteins. *Nature* 608: 618–625

Aukerman MJ, Sakai H (2003) Regulation of flowering time and floral organ identity by a MicroRNA and its APETALA2-like target genes. *Plant Cell* 15: 2730–2741

Baek D, Villen J, Shin C, Camargo FD, Gygi SP, Bartel DP (2008) The impact of microRNAs on protein output. *Nature* 455: 64–71

Bartel DP (2018) Metazoan MicroRNAs. *Cell* 173: 20–51

Becker WR, Ober-Reynolds B, Jouravleva K, Jolly SM, Zamore PD, Greenleaf WJ (2019) High-throughput analysis reveals rules for target RNA binding and cleavage by AGO2. *Mol Cell* 75: 741–755.e11

Bibel B, Elkayam E, Silletti S, Komives EA, Joshua-Tor L (2022) Target binding triggers hierarchical phosphorylation of human Argonaute-2 to promote target release. *Elife* 11: e76908

Bohmert K, Camus I, Bellini C, Bouchez D, Caboche M, Benning C (1998) AGO1 defines a novel locus of Arabidopsis controlling leaf development. *EMBO J* 17: 170–180

Borges F, Martienssen RA (2015) The expanding world of small RNAs in plants. *Nat Rev Mol Cell Biol* 16: 727–741

Brodersen P, Sakvarelidze-Achard L, Bruun-Rasmussen M, Dunoyer P, Yamamoto YY, Sieburth L, Voinnet O (2008) Widespread translational inhibition by plant miRNAs and siRNAs. *Science* 320: 1185–1190

Castellanos-Gonzalez A, Perry N, Nava S, White AC Jr (2016) Preassembled single-stranded RNA-Argonaute complexes: a novel method to silence genes in cryptosporidium. *J Infect Dis* 213: 1307–1314

Chandradoss SD, Schirle NT, Szczepaniak M, MacRae IJ, Joo C (2015) A dynamic search process underlies MicroRNA targeting. *Cell* 162: 96–107

Cheloufi S, Dos Santos CO, Chong MM, Hannon GJ (2010) A dicer-independent miRNA biogenesis pathway that requires ago catalysis. *Nature* 465: 584–589

Chen X (2004) A microRNA as a translational repressor of APETALA2 in *Arabidopsis* flower development. *Science* 303: 2022–2025

Chen GR, Sive H, Bartel DP (2017) A seed mismatch enhances Argonaute2-catalyzed cleavage and partially rescues severely impaired cleavage found in fish. *Mol Cell* 68: 1095–1107.e5

Cifuentes D, Xue H, Taylor DW, Patnode H, Mishima Y, Cheloufi S, Ma E, Mane S, Hannon GJ, Lawson ND *et al* (2010) A novel miRNA processing pathway independent of dicer requires Argonaute2 catalytic activity. *Science* 328: 1694–1698

Deerberg A, Willkomm S, Restle T (2013) Minimal mechanistic model of siRNA-dependent target RNA slicing by recombinant human Argonaute 2 protein. *Proc Natl Acad Sci USA* 110: 17850–17855

Elbashir SM, Harborth J, Lendeckel W, Yalcin A, Weber K, Tuschl T (2001a) Duplexes of 21-nucleotide RNAs mediate RNA interference in cultured mammalian cells. *Nature* 411: 494–498

Elbashir SM, Lendeckel W, Tuschl T (2001b) RNA interference is mediated by 21- and 22-nucleotide RNAs. *Genes Dev* 15: 188–200

Emsley P, Lohkamp B, Scott WG, Cowtan K (2010) Features and development of coot. *Acta Crystallogr D Biol Crystallogr* 66: 486–501

Friedman RC, Farh KK, Burge CB, Bartel DP (2009) Most mammalian mRNAs are conserved targets of microRNAs. *Genome Res* 19: 92–105

Gandikota M, Birkenbihl RP, Hohmann S, Cardon GH, Saedler H, Huijser P (2007) The miRNA156/157 recognition element in the 3′ UTR of the Arabidopsis SBP box gene SPL3 prevents early flowering by translational inhibition in seedlings. *Plant J* 49: 683–693

Golden RJ, Chen B, Li T, Braun J, Manjunath H, Chen X, Wu J, Schmid V, Chang TC, Kopp F *et al* (2017) An Argonaute phosphorylation cycle promotes microRNA-mediated silencing. *Nature* 542: 197–202

Haley B, Zamore PD (2004) Kinetic analysis of the RNAi enzyme complex. *Nat Struct Mol Biol* 11: 599–606

Iwakawa HO, Tomari Y (2013) Molecular insights into microRNA-mediated translational repression in plants. *Mol Cell* 52: 591–601

Jackson AL, Linsley PS (2010) Recognizing and avoiding siRNA off-target effects for target identification and therapeutic application. *Nat Rev Drug Discov* 9: 57–67

Jonas S, Izaurralde E (2015) Towards a molecular understanding of microRNA-mediated gene silencing. *Nat Rev Genet* 16: 421–433

Jouravleva K, Vega-Badillo J, Zamore PD (2022) Principles and pitfalls of high-throughput analysis of microRNA-binding thermodynamics and kinetics by RNA bind-n-seq. *Cell Rep Methods* 2: 100185

Kabsch W (2010) Xds. *Acta Crystallogr D Biol Crystallogr* 66: 125–132

Klum SM, Chandradoss SD, Schirle NT, Joo C, MacRae IJ (2017) Helix-7 in Argonaute2 shapes the microRNA seed region for rapid target recognition. *EMBO J* 37: 75–88

Lewis BP, Burge CB, Bartel DP (2005) Conserved seed pairing, often flanked by adenosines, indicates that thousands of human genes are microRNA targets. *Cell* 120: 15–20

Li S, Liu L, Zhuang X, Yu Y, Liu X, Cui X, Ji L, Pan Z, Cao X, Mo B *et al* (2013) MicroRNAs inhibit the translation of target mRNAs on the endoplasmic reticulum in *Arabidopsis*. *Cell* 153: 562–574

 © 2023 The Authors

Li J, Wu C, Wang W, He Y, Elkayam E, Joshua-Tor L, Hammond PT (2018) Structurally modulated codelivery of siRNA and Argonaute 2 for enhanced RNA interference. *Proc Natl Acad Sci USA* 115: E2696–E2705

Liu J, Carmell MA, Rivas FV, Marsden CG, Thomson JM, Song JJ, Hammond SM, Joshua-Tor L, Hannon GJ (2004) Argonaute2 is the catalytic engine of mammalian RNAi. *Science* 305: 1437–1441

Liu Q, Wang F, Axtell MJ (2014) Analysis of complementarity requirements for plant microRNA targeting using a Nicotiana benthamiana quantitative transient assay. *Plant Cell* 26: 741–753

Llave C, Xie Z, Kasschau KD, Carrington JC (2002) Cleavage of scarecrow-like mRNA targets directed by a class of *Arabidopsis* miRNA. *Science* 297: 2053–2056

Meister G, Landthaler M, Patkaniowska A, Dorsett Y, Teng G, Tuschl T (2004) Human Argonaute2 mediates RNA cleavage targeted by miRNAs and siRNAs. *Mol Cell* 15: 185–197

Ober-Reynolds B, Becker WR, Jouravleva K, Jolly SM, Zamore PD, Greenleaf WJ (2022) High-throughput biochemical profiling reveals functional adaptation of a bacterial Argonaute. *Mol Cell* 82: 1329–1342.e8

Parker JS, Parizotto EA, Wang M, Roe SM, Barford D (2009) Enhancement of the seed-target recognition step in RNA silencing by a PIWI/MID domain protein. *Mol Cell* 33: 204–214

Pinzon N, Li B, Martinez L, Sergeeva A, Presumey J, Apparailly F, Seitz H (2017) microRNA target prediction programs predict many false positives. *Genome Res* 27: 234–245

Quevillon Huberdeau M, Zeitler DM, Hauptmann J, Bruckmann A, Fressigne L, Danner J, Piquet S, Strieder N, Engelmann JC, Jannot G *et al* (2017) Phosphorylation of Argonaute proteins affects mRNA binding and is essential for microRNA-guided gene silencing in vivo. *EMBO J* 36: 2088–2106

van Rij RP, Saleh MC, Berry B, Foo C, Houk A, Antoniewski C, Andino R (2006) The RNA silencing endonuclease Argonaute 2 mediates specific antiviral immunity in Drosophila melanogaster. *Genes Dev* 20: 2985–2995

Rivas FV, Tolia NH, Song JJ, Aragon JP, Liu J, Hannon GJ, Joshua-Tor L (2005) Purified Argonaute2 and an siRNA form recombinant human RISC. *Nat Struct Mol Biol* 12: 340–349

Salomon WE, Jolly SM, Moore MJ, Zamore PD, Serebrov V (2015) Single-molecule imaging reveals that Argonaute reshapes the binding properties of its nucleic acid guides. *Cell* 162: 84–95

Schirle NT, MacRae IJ (2012) The crystal structure of human Argonaute2. *Science* 336: 1037–1040

Schirle NT, Sheu-Gruttadauria J, MacRae IJ (2014) Structural basis for microRNA targeting. *Science* 346: 608–613

Schnettler E, Tykalova H, Watson M, Sharma M, Sterken MG, Obbard DJ, Lewis SH, McFarlane M, Bell-Sakyi L, Barry G *et al* (2014) Induction and suppression of tick cell antiviral RNAi responses by tick-borne flaviviruses. *Nucleic Acids Res* 42: 9436–9446

Seitz H (2017) Issues in current microRNA target identification methods. *RNA Biol* 14: 831–834

Seok H, Lee H, Jang ES, Chi SW (2018) Evaluation and control of miRNA-like off-target repression for RNA interference. *Cell Mol Life Sci* 75: 797–814

Sheu-Gruttadauria J, MacRae IJ (2018) Phase transitions in the assembly and function of human miRISC. *Cell* 173: 946–957.e16

Sheu-Gruttadauria J, Pawlica P, Klum SM, Wang S, Yario TA, Schirle Oakdale NT, Steitz JA, MacRae IJ (2019a) Structural basis for target-directed MicroRNA degradation. *Mol Cell* 75: 1243–1255.e7

Sheu-Gruttadauria J, Xiao Y, Gebert LF, MacRae IJ (2019b) Beyond the seed: structural basis for supplementary microRNA targeting by human Argonaute2. *EMBO J* 38: e101153

Syed YY (2021) Givosiran: a review in acute hepatic porphyria. *Drugs* 81: 841–848

Tang G, Reinhart BJ, Bartel DP, Zamore PD (2003) A biochemical framework for RNA silencing in plants. *Genes Dev* 17: 49–63

Vaucheret H (2008) Plant ARGONAUTES. *Trends Plant Sci* 13: 350–358

Wang XH, Aliyari R, Li WX, Li HW, Kim K, Carthew R, Atkinson P, Ding SW (2006) RNA interference directs innate immunity against viruses in adult drosophila. *Science* 312: 452–454

Wang Y, Sheng G, Juranek S, Tuschl T, Patel DJ (2008) Structure of the guide-strand-containing argonaute silencing complex. *Nature* 456: 209–213

Wee LM, Flores-Jasso CF, Salomon WE, Zamore PD (2012) Argonaute divides its RNA guide into domains with distinct functions and RNA-binding properties. *Cell* 151: 1055–1067

Weng Y, Xiao H, Zhang J, Liang XJ, Huang Y (2019) RNAi therapeutic and its innovative biotechnological evolution. *Biotechnol Adv* 37: 801–825

Wilkins C, Dishongh R, Moore SC, Whitt MA, Chow M, Machaca K (2005) RNA interference is an antiviral defence mechanism in Caenorhabditis elegans. *Nature* 436: 1044–1047

Winn MD, Ballard CC, Cowtan KD, Dodson EJ, Emsley P, Evans PR, Keegan RM, Krissinel EB, Leslie AG, McCoy A *et al* (2011) Overview of the CCP4 suite and current developments. *Acta Crystallogr D Biol Crystallogr* 67: 235–242

Wynant N, Santos D, Vanden Broeck J (2017) The evolution of animal Argonautes: evidence for the absence of antiviral AGO Argonautes in vertebrates. *Sci Rep* 7: 9230

Xiao Y, MacRae IJ (2022) The molecular mechanism of microRNA duplex selectivity of Arabidopsis ARGONAUTE10. *Nucleic Acids Res* 50: 10041–10052

Xiao Y, Maeda S, Otomo T, MacRae IJ (2022) Structural basis for RNA slicing by a plant Argonaute. *bioxRiv* https://doi.org/10.1101/2022.07.15.500266 [PREPRINT]

License: This is an open access article under the terms of the Creative Commons Attribution License, which permits use, distribution and reproduction in any medium, provided the original work is properly cited.

Expanded View Figures

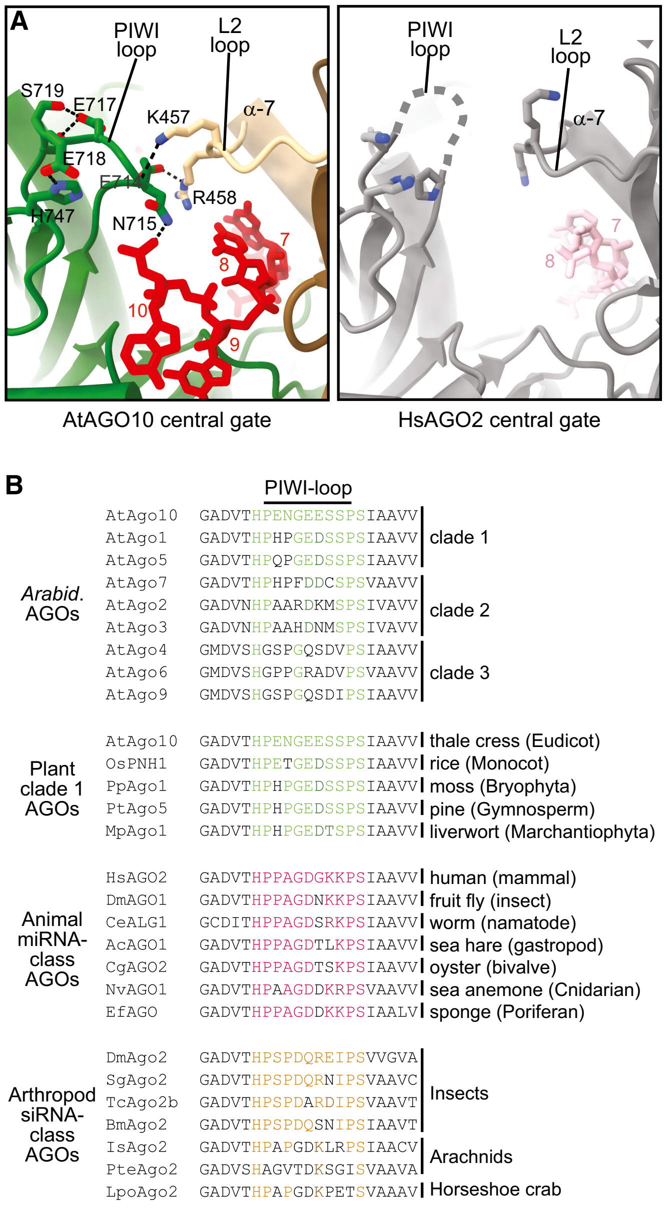

Figure EV1. Comparison of PIWI loops in diverse AGOs.

A Close-up views of PIWI-loop and L2-loop connections in AtAGO10 (PDB 7SVA) and HsAGO2 (PDB 4OLA) structures.

B Alignment of PIWI-loop sequences from diverse eukaryotes.

© 2023 The Authors

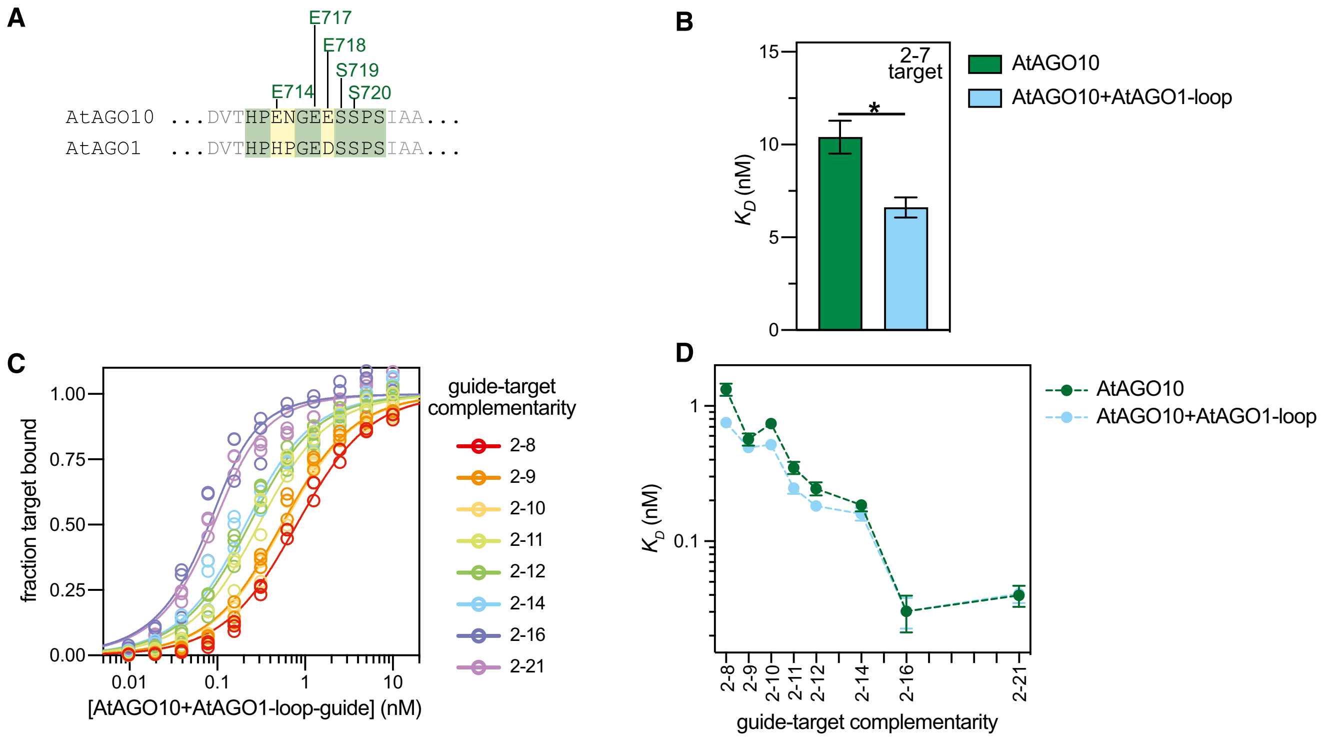

Figure EV2. PIWI loops of AtAGO1 and AtAGO10 are functionally similar.

A Detailed comparison of PIWI-loop sequences in AtAGO10 and AtAGO1.

B Dissociation constants for wild-type AtAGO10 and a mutant AtAGO10 bearing the PIWI-loop sequence from AtAGO1 (AtAGO10 + AtAGO10-loop) binding to a target RNA with complementarity to the miRNA seed region (2–7).

C Fraction target RNAs bound versus [AtAGO1 + AtAGO1-loop]. Guide and target RNAs are shown in Fig 2A.

D K_D values of wild-type AtAGO10 and AtAGO10 + AtAGO1-loop mutant for various target RNAs.

Data information: Data points in panel (C) are from $n = 3$ independent technical trials. Data in panels (B and D) represent best-fit K_D values from $n = 3$ technical trials. Error bars indicate s.e.m. Unpaired *t*-test *P*-value: *0.0219. No data points were excluded from the analyses.

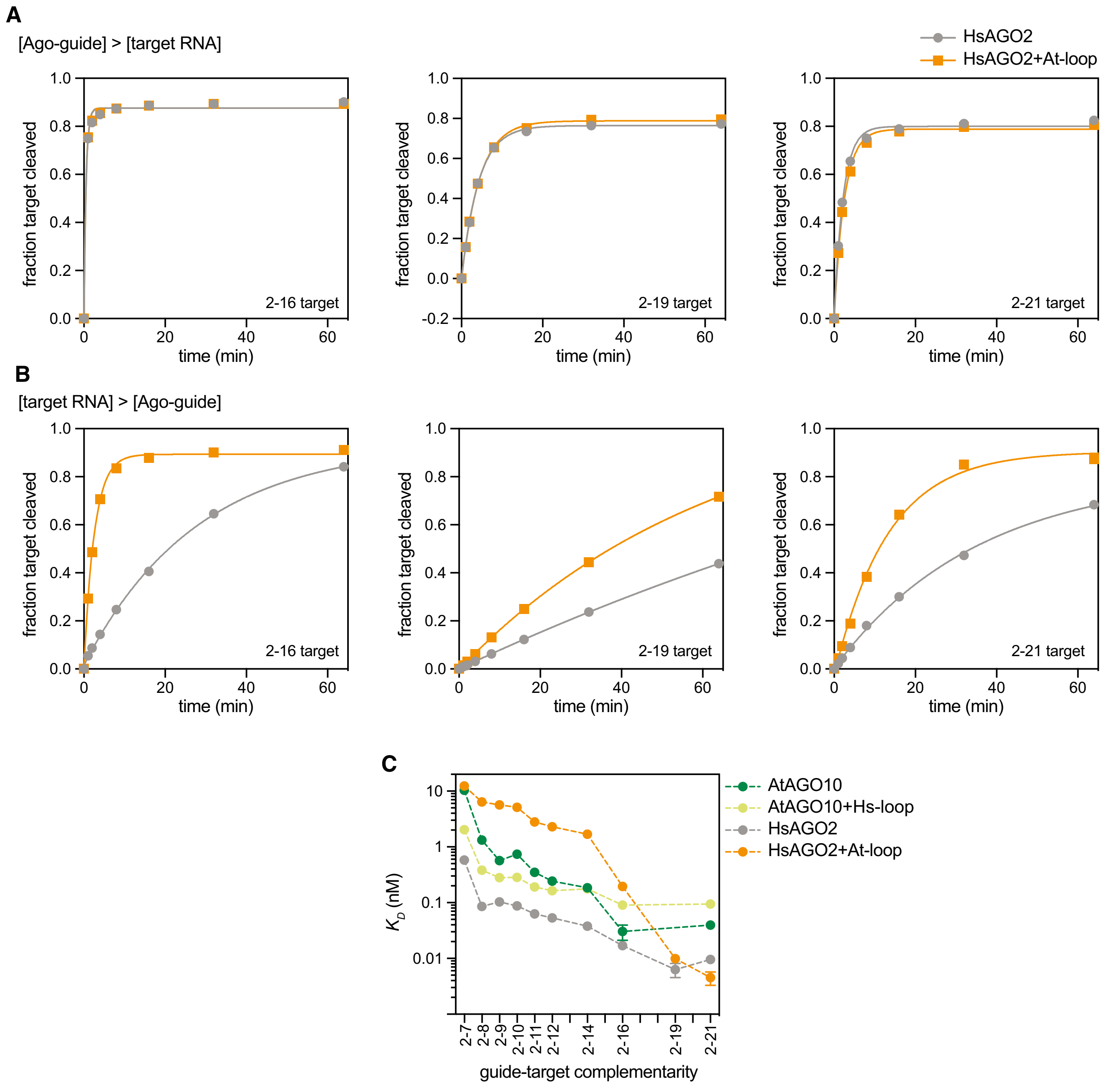

Figure EV3. Detailed comparison of HsAGO2 and HsAGO2 + At-loop biochemical properties.

A Fraction of target RNAs cleaved versus time when cleavage reaction is limited by target RNA ([Ago-guide] = 10 nM, [target RNA] = 2 nM).

B Fraction of target RNAs cleaved versus time when cleavage reaction is limited by Ago-guide ([Ago-guide] = 1 nM, [target RNA] = 5 nM).

C Comparison of K_D values of HsAGO2, HsAGO2 + At-loop, AtAGO10, and AtAGO10 + Hs-loop for various target RNAs.

Data information: Data points in panels (A and B) are values from $n = 1$ experimental trials. Data points in panel (C) are the best-fit K_D values from $n = 3$ independent technical trials, each with ≥ 11 data points. Error bars indicate s.e.m. No data points were excluded from the analyses.

 © 2023 The Authors
