# Supplementary material for: A tiny loop in the Argonaute PIWI domain tunes small RNA seed strength
Source: EMBO Rep. 2023 Apr 21;24(6):e55806. doi: 10.15252/embr.202255806 (PMC10240194; doi:10.15252/embr.202255806)
Supplement: Supplementary file 6 [file EMBR-24-e55806-s004.pdf]

EMBO reports

A tiny loop in the Argonaute PIWI domain tunes small RNA seed strength

Yao Xiao, TingYu Liu, and Ian MacRae
DOI: 10.15252/embr.202255806

Corresponding author(s): Ian MacRae (macrae@scripps.edu)

Review Timeline:

Submission Date:	18th Jul 22
Editorial Decision:	23rd Aug 22
Revision Received:	21st Mar 23
Editorial Decision:	29th Mar 23
Revision Received:	29th Mar 23
Accepted:	5th Apr 23

Editor: Esther Schnapp

Transaction Report:

(Note: With the exception of the correction of typographical or spelling errors that could be a source of ambiguity, letters and reports are not edited. Depending on transfer agreements, referee reports obtained elsewhere may or may not be included in this compilation. Referee reports are anonymous unless the Referee chooses to sign their reports.)

1st Editorial Decision **23rd Aug 2022**

Dear Ian,

Thank you for the submission of your manuscript to EMBO reports. We have now received the comments from 2 referees, one report is pasted below and one is attached as pdf file to this email.

As you will see, the referees acknowledge that the findings are interesting and novel. They only raise rather minor concerns that should all be addressed, except for point 5 by referee 2 that is optional.

I would thus like to invite you to revise your manuscript with the understanding that the referee concerns must be fully addressed and their suggestions taken on board. Please address all referee concerns in a complete point-by-point response. Acceptance of the manuscript will depend on a positive outcome of a second round of review. It is EMBO reports policy to allow a single round of major revision only and acceptance or rejection of the manuscript will therefore depend on the completeness of your responses included in the next, final version of the manuscript.

We realize that it is difficult to revise to a specific deadline. In the interest of protecting the conceptual advance provided by the work, we recommend a revision within 3 months (23rd Nov 2022). Please discuss the revision progress ahead of this time with the editor if you require more time to complete the revisions.

You can either publish the study as a short report or as a full article. For short reports, the revised manuscript should not exceed 27,000 characters (including spaces but excluding materials & methods and references) and 5 main plus 5 expanded view figures. The results and discussion sections must further be combined, which will help to shorten the manuscript text by eliminating some redundancy that is inevitable when discussing the same experiments twice. For a normal article there are no length limitations, but it should have more than 5 main figures and the results and discussion sections must be separate.

IMPORTANT NOTE: we perform an initial quality control of all revised manuscripts before re-review. Your manuscript will FAIL this control and the handling will be DELAYED if the following APPLIES:
1) A data availability section providing access to data deposited in public databases is missing. If you have not deposited any data, please add a sentence to the data availability section that explains that.
2) Your manuscript contains statistics and error bars based on n=2. Please use scatter blots in these cases. No statistics should be calculated if n=2.

When submitting your revised manuscript, please carefully review the instructions that follow below. Failure to include requested items will delay the evaluation of your revision.

1) a .docx formatted version of the manuscript text (including legends for main figures, EV figures and tables). Please make sure that the changes are highlighted to be clearly visible.

2) individual production quality figure files as .eps, .tif, .jpg (one file per figure). See https://wol-prod-cdn.literatumonline.com/pb-assets/embo-site/EMBOPress_Figure_Guidelines_061115-1561436025777.pdf for more info on how to prepare your figures.

3) We replaced Supplementary Information with Expanded View (EV) Figures and Tables that are collapsible/expandable online. A maximum of 5 EV Figures can be typeset. EV Figures should be cited as 'Figure EV1, Figure EV2" etc... in the text and their respective legends should be included in the main text after the legends of regular figures.

- For the figures that you do NOT wish to display as Expanded View figures, they should be bundled together with their legends in a single PDF file called *Appendix*, which should start with a short Table of Content. Appendix figures should be referred to in the main text as: "Appendix Figure S1, Appendix Figure S2" etc. See detailed instructions regarding expanded view here: <https://www.embopress.org/page/journal/14693178/authorguide#expandedview>

- Additional Tables/Datasets should be labeled and referred to as Table EV1, Dataset EV1, etc. Legends have to be provided in a separate tab in case of .xls files. Alternatively, the legend can be supplied as a separate text file (README) and zipped together with the Table/Dataset file.

4) a .docx formatted letter INCLUDING the reviewers' reports and your detailed point-by-point responses to their comments. As part of the EMBO Press transparent editorial process, the point-by-point response is part of the Review Process File (RPF), which will be published alongside your paper.

5) a complete author checklist, which you can download from our author guidelines <https://www.embopress.org/page/journal/14693178/authorguide>. Please insert information in the checklist that is also reflected in the manuscript. The completed author checklist will also be part of the RPF.

6) Please note that all corresponding authors are required to supply an ORCID ID for their name upon submission of a revised

manuscript (<https://orcid.org/>). Please find instructions on how to link your ORCID ID to your account in our manuscript tracking system in our Author guidelines <https://www.embopress.org/page/journal/14693178/authorguide#authorshipguidelines>

7) Before submitting your revision, primary datasets produced in this study need to be deposited in an appropriate public database (see https://www.embopress.org/page/journal/14693178/authorguide#datadeposition). Please remember to provide a reviewer password if the datasets are not yet public. The accession numbers and database should be listed in a formal "Data Availability" section placed after Materials & Method (see also https://www.embopress.org/page/journal/14693178/authorguide#datadeposition). Please note that the Data Availability Section is restricted to new primary data that are part of this study. * Note - All links should resolve to a page where the data can be accessed. *
If your study has not produced novel datasets, please mention this fact in the Data Availability Section.

8) We would also encourage you to include the source data for figure panels that show essential data. Numerical data should be provided as individual .xls or .csv files (including a tab describing the data). For blots or microscopy, uncropped images should be submitted (using a zip archive if multiple images need to be supplied for one panel). Additional information on source data and instruction on how to label the files are available at <https://www.embopress.org/page/journal/14693178/authorguide#sourcedata>.

9) Our journal also encourages inclusion of *data citations in the reference list* to directly cite datasets that were re-used and obtained from public databases. Data citations in the article text are distinct from normal bibliographical citations and should directly link to the database records from which the data can be accessed. In the main text, data citations are formatted as follows: "Data ref: Smith et al, 2001" or "Data ref: NCBI Sequence Read Archive PRJNA342805, 2017". In the Reference list, data citations must be labeled with "[DATASET]". A data reference must provide the database name, accession number/identifiers and a resolvable link to the landing page from which the data can be accessed at the end of the reference. Further instructions are available at https://www.embopress.org/page/journal/14693178/authorguide#referencesformat

10) Regarding data quantification (see Figure Legends: https://www.embopress.org/page/journal/14693178/authorguide#figureformat)

The following points must be specified in each figure legend:

- the name of the statistical test used to generate error bars and P values,

- the number (n) of independent experiments (please specify technical or biological replicates) underlying each data point,

- the nature of the bars and error bars (s.d., s.e.m.),

- If the data are obtained from n {less than or equal to} 2, use scatter blots showing the individual data points.

Discussion of statistical methodology can be reported in the materials and methods section, but figure legends should contain a basic description of n, P and the test applied.

- Please also include scale bars in all microscopy images.

11) The journal requires a statement specifying whether or not authors have competing interests (defined as all potential or actual interests that could be perceived to influence the presentation or interpretation of an article). In case of competing interests, this must be specified in your disclosure statement. Further information: https://www.embopress.org/competing-interests

We would also welcome the submission of cover suggestions, or motifs to be used by our Graphics Illustrator in designing a cover.

As part of the EMBO publication's Transparent Editorial Process, EMBO reports publishes online a Review Process File (RPF) to accompany accepted manuscripts. This File will be published in conjunction with your paper and will include the referee reports, your point-by-point response and all pertinent correspondence relating to the manuscript.

You are able to opt out of this by letting the editorial office know (emboreports@embo.org). If you do opt out, the Review Process File link will point to the following statement: "No Review Process File is available with this article, as the authors have chosen not to make the review process public in this case."

I look forward to seeing a revised form of your manuscript when it is ready. Please use this link to submit your revision: https://embor.msubmit.net/cgi-bin/main.plex

Best,
Esther

Esther Schnapp, PhD
Senior Editor
EMBO reports

Referee #1:

In this excellent small study, the authors use recently obtained structures of a plant ARGONAUTE protein as a basis to design experiments to obtain answers to a question of substantial importance for our understanding of the mechanistic basis of RNA interference phenomena: Why is it that base pairing between small RNA and target limited to the so-called seed region (nucleotides 2-7/8 of the small RNA) is sufficient for target regulation in metazoans, but not in plants? This question is all the more pertinent now that we know with certainty what was already expected from sequence conservation and Alphafold predictions: the overall structures of mammalian and plant Argonaute proteins are highly similar.

The authors first use rigorous experimentation to show directly that AGO10 - the plant AGO protein used for structural studies published elsewhere - binds with ~20-fold lower affinity compared to human Ago2 (Hs-Ago2) to a target RNA exhibiting seed complementarity. They then notice that a small loop in the Piwi domain adopts an ordered conformation in AGO10, but not in Hs-Ago2, including several interactions to the important helix-7 that is well-described in previous studies to influence seed pairing between guide and target RNA. The remainder of the paper uses, in the main, swapping experiments, in which the small Piwi-loop of Hs-Ago2 is inserted into AGO10, or the plant Piwi-loop (from AGO10 or AGO1) is inserted into Hs-Ago2, and the biochemical and/or silencing properties in cells are analysed. The experiments convincingly demonstrate that the major mechanistic basis of the different biochemical properties of plant and human miRNA-binding AGO proteins rely on the small Piwi-loop in the central cleft of the proteins. Specifically, the authors demonstrate that
(i) AGO10 harboring the Hs-Ago2 loop shows dramatically enhanced affinity to seed targets, including, importantly, a reduced dynamic range of binding affinity as a function of degree of small RNA:target complementarity
(ii) the majority of AGO10 molecules exhibit fast seed target-release kinetics, but in the presence of the Hs-Ago2 loop, the entire population switches to slow release.
(iii) Hs-Ago2 harboring the AGO10 loop has a structure with a well-defined conformation of the AGO10-loop, exhibits reduced affinity for seed targets, and, correspondingly, a dramatically enhanced dynamic range of binding affinity as a function of degree of small RNA:target complementarity
(iv) Hs-Ago2-AGO10 loop variant shows enhanced catalytic turnover in multi-turnover settings (but not in single-turnover conditions), consistent with more rapid target release
(v) the Hs-Ago2-AGO10 loop variant gives rise to substantially increased on-target/off-target ratios in experimental RNAi with siRNA duplexes and plasmid borne reporters in cell culture.

The experiments and well executed, results are surprisingly clear, and the interpretations of the results are well supported. More in vivo experiments, particularly on the plant side, would be desirable, but can be performed by other researchers following the publication of the results contained in this report. My suggestions for improvement of the current manuscript prior to publication are relatively minor, and focus on the crafting of the manuscript rather than the work itself that I find impressive and elegant.

(1) In the abstract and Introduction, I think the authors should take more care in how they describe the biological consequences of the clearly different properties in small RNA-guided targeting between plant and animal AGO proteins. First, it is not unequivocally established that "animals use miRNAs to sculpt the transcriptome" although some researchers advance this view. Please remember that although many mRNAs are targeted by a single miRNA in vivo, exactly what the biological consequence of this is, is more difficult to determine: many important effects of miRNAs in development can be explained by misregulation of only a few targets as demonstrated by multiple genetic studies in C. elegans and Drosophila, and there is good evidence that the multitude of conserved mRNA targets may serve to regulate miRNA activity rather than "sculpting the transcriptome" (Pinzon et al. (2016), Genome Research, https://genome.cshlp.org/content/early/2016/11/07/gr.205146.116).
Similarly, the authors should note that to date, not a single study reporting on unbiased mapping of AGO1/miRNA binding sites throughout a plant transcriptome has been published, thus making it not fully established how miRNAs actually behave in vivo in plants.
Notwithstanding the need to amend to oversimplification of some of these sections, the case of importance for the present study is clear enough: the targeting properties of animals and plant RISC are different!

(2) The literature used to support the statement that seed complementarity is not sufficient for miRNA targeting in plants should include the thorough in vivo work on this matter by the Axtell lab (Liu et al., (2014), Plant Cell, https://academic.oup.com/plcell/article/26/2/741/6097966).
(3) The treatment of AGO10 and AGO1 in the Introduction is not satisfactory. Although it is true that AGO10 is a close paralogue of the main miRISC component in plants, AGO1, it is not true to summarize their in vivo functions by the statement "with overlapping functions in vivo". This genetic interpretation is based on very early mutant studies that showed that ago1/ago10 double knockout mutants are embryonically lethal in contrast to either single mutant, although each of the single mutants has

severe developmental defects in the genetic background analyzed at the time. We now know that ago1 shows these defects because of lack of miRNA-guided silencing, but ago10 can show defects because of enhanced AGO1-miR166 guided silencing. The "overlapping function" was a fair genetic interpretation in 1999, but is misleading to pass on to readers in 2022. Again, the authors need to take a bit more care not to oversimplify which should be easily possible while maintaining the clear flow of the manuscript and emphasizing the most important points for their study.
(4) The authors could consider re-labeling panels in Fig. 1 so that the close-ups become B and are referred to at first use. It is a bit hard to see the point of identical seed arrangement from the full structure views in A.
(5) The authors could consider including a comment on the E714H substitution comparing AGO10 to AGO1 in relation to the slightly behaviors of the insertions of these two proteins, in particular because interactions with E714 are highlighted.
(6) The authors could consider helping the reader with a separate illustration to guide the paragraph describing the "Structural model for miRNA targeting selectivity in plants". I found the reference to Fig. 1 a bit minimalistic in this regard.
(7) The sentence "the N603 side chain is within H-bonding distance of the main chain amine of K355..." requires correction. Is it backbone amide of K355 or side chain amine?
(8) The literature used to support the statement that mulitturnover rates are largely dictated by rate of product release is incomplete and should, as a minimum, also include the stop-flow studies reported by Deerberg et al. (2013) PNAS 110(44), 17850-5. https://www.pnas.org/doi/10.1073/pnas.1217838110
(9) The discussion focuses very much on the, admittedly, very important result that selectivity and efficiency of RNAi in humans can be enhanced based on the findings in here. The results are also of high relevance for plant biology, however, and it would perhaps be relevant to discuss some of this? One suggestion would be to include a comment on the fact that 15% of the AGO10 population shows slow release kinetics despite the presence of the Piwi-loop. Could one imagine that interaction with co-factors may increase this percentage such that seed-type matching could be regulated in plants, perhaps with ties to the many reports on translational repression by miRNAs that are highly complementary to their targets?
(10) The language and writing style could benefit from a bit of improvement, although the manuscript reads fine overall.

To sum up, I support publication of the manuscript without the need for further experimentation, but with some work put into improvement of the crafting of the manuscript. I would like to congratulate the authors for an interesting and well-executed study!

Referee #2

The differences between animal and plant AGO proteins pose many important evolutionary, biological, and biochemical questions. This study solves a longstanding question in Argonaute enzymology: why do plant AGOs regulate their targets by cleavage ("siRNA-like) rather than the mechanism used by animals, in which AGOs bind a short, seed-matched sequence, then recruit other cellular components to reduce translation or stability of the target mRNA. The data are rigorous and clearly presented, and the manuscript is a pleasure to read. Even my major comments are fairly minor. I recommend publication of a revised manuscript that addresses my concerns below. (I would like to note that this study is more appropriate for *EMBO J.* than for *EMBO Reports.*)

Major Comments

(1) Page 7: How was k_{cat} determined? For single-turnover reactions displaying burst kinetics,

$$y(x) = \mathrm{E}_0 \times \left[(k_2/(k_2 + k_3))^2 \times \left(1 - e^{-t \times (k_2 + k_3)}\right) + t \times k_2 \times k_3/(k_2 + k_3)\right],$$

and the time-dependence of product formation corresponded to a pre-steady-state exponential burst ($k_{burst} = k_2 + k_3$) followed by a linear steady-state phase, described by k_{cat}, where $k_{cat} = k_2 \times k_3/(k_2 + k_3)$. Please provide additional details for how both single- and multiple-turnover cleavage data was analyzed and whether E_0 was fitted, measured via a binding assay, or assumed to be 100% active. Using this equation will allow the authors to determine for graphs like those in Figure S2D (e.g., bottom, gray line) the mix of burst and multiple-turnover rates that contribute to the apparent absence of a burst.

(2) Is the 1.1–1.2-fold repression (green in Figure 5B) statistically different from no repression? If the error bars show standard deviation, they are surprisingly small. Was the error of the unrepressed control propagated through the error of the experiment using standard methods?

For $z = c\frac{x}{y}$ error is propagated as $\frac{\Delta z}{z} = \left[\left(\frac{\Delta x}{x}\right)^2 + \left(\frac{\Delta y}{y}\right)^2\right]^{1/2}$.

(3) I understand the intent of the chart in Figure 5C, but from an evolutionary perspective, repression of the seed-matched reporter by the siRNA duplex or

HsAGO2 RISC is on-target, not off-target. The siRNA is being used as a bona fide miRNA in an animal lacking an RNAi pathway. Perhaps a less normative nomenclature could be used, such as miRNA-like vs. siRNA-like repression?

(4) An unpaired t-test can't formally be used to make pairwise comparisons in an experiment in which more than one hypothesis is tested (here, the hypotheses are is blue different from green and is red different from green). Typically, ANOVA followed by a post-hoc test is used.

(5) If it isn't too much work, it would be useful to know whether HsAGO2+At-loop shows superior target silencing compared to fly Ago2, which evolved to mediate RNAi and is rarely loaded with miRNAs in vivo.

(6) Can phylogenetic analyses including other plant and animal AGOs help explain whether the animal or the plant PIWI loop is the likely ancestral state?

(7) Could the more "rigid" loop in plant AGOs help them avoid cleaving targets with limited complementarity? Comparing AtAGO10-Hs and AtAGO10 cleavage pairing requirements could provide the answer.

(8) Can the reduction in the range of affinities to just 4-fold upon introduction of Hs-loop into AtAGO10 be explained by a stronger affinity of AtAGO10-Hs for the canonical short seed and AtAGO10-Hs avoiding central pairing just like HsAGO2?

<u>Minor Comments</u>

Abstract: (1) promiscuity implies lack of specificity; HsAGO2-bound miRNAs are highly specific, in that they bind perfect seed matches much more strongly than mismatched sites. There are simply more seed matches in the transcriptome because the site length is short compared to plant miRNAs. Sequence-specific RNA-binding proteins often recognize sites of the same length as a miRNA seed match. (2) Not all animals use miRNAs to repress hundreds of targets. See for example, Moran, Y. et al. Cnidarian microRNAs frequently regulate targets by cleavage. *Genome Res* **24**, 651-663 (2014). Plant and animal AGOs are not more or less promiscuous, discerning, or stringent, they use different site lengths for target recognition. Moreover, the binding mechanisms of fly AGO2 and of animal PIWI proteins strongly suggest that HsAGO2 is more, not less, stringent than AGOs that recognize long target sites, in that it tolerates fewer mismatches in a single site. See also Herschlag, D. Implications of ribozyme kinetics for targeting the cleavage of specific RNA molecules in vivo: more isn't always better. *Proc Natl Acad Sci U S A* **88**, 6921-6925 (1991).

Introduction: (1) The majority of the world's animals are arthropods, most of which likely use Ago2 (Ago1 is the arthropod homolog of AGO2) and siRNAs as

an antiviral defense. It is worthwhile mentioning this (and commenting on the PIWI loop length in that protein). Ago2 seems to be more like AGO2 than plant AGOs. Does this make structural sense? This is especially important given the discussion about therapeutic siRNAs in animals lacking a formal RNAi pathway (top of page 3). (2) I think 2–3-fold is much greater than the typical effect of miRNA-binding on the steady-state abundance of an mRNA target for HsAGO2. If the typical effect were that large, no one would need to use CDF plots to see it. (3) There is a growing body of evidence that animal miRNAs do not sculpt the genome. Both views should be presented. E.g., Pinzón, N. et al. microRNA target prediction programs predict many false positives. *Genome Res* **27**, 234-245 (2017) and Seitz, H. Issues in current microRNA target identification methods. *RNA Biol* **14**, 831-834 (2017). (4) What clinical trial has failed as a consequence of miRNA-like off-target effects? To the best of my knowledge, it is not possible to achieve a sufficiently high intracellular concentration of drug in RISC in the target tissue to mediate such effects. (I doubt that there would be five FDA-approved siRNA drugs in clinical use today if such effects really happened.)

Results: (1) When percent active enzyme is used in calculating dissociation constants, animal RISCs whose seed sequence fully complementary to a target site have K_D values in the pM, not nM range. E.g., this manuscript; Jouravleva et al. (*Cell Reports Methods* 2022); Salomon et al. (*Cell* 2015); Becker et al. (*Mol Cell* 2019).

Figure 1B: it would be helpful to include the guide and target sequences in the figure.

Figure 4D, right panel: should *K* (equilibrium constant) be *k* (rate constant)? Also, should the callout " Fig. 4E,F" on page 7 be 4D?

Wee et al. (*Cell* 2012) used mouse, not human AGO2 (they are nearly identical, of course).

Thermus thermophilus TtAgo behaves like an animal AGO at 37°C and like a plant AGO at 55°C (Smith, C. S. et al. An automated Bayesian pipeline for rapid analysis of single-molecule binding data. *Nat Commun* **10**, 272 (2019). Can your observations help explain why?

Perhaps the title is over broad given that only two AGO proteins were studied (HsAGO2 and AtAGO10)?

Response to referees

> We are grateful to both referees for using their time and energy to prepare these thoughtful reviews of our work. We did our best to incorporate your suggestions and feel the revised manuscript is more accurate and inclusive of models in the field, conveying our ideas more accurately, and has strengthened conclusions through the addition of more experimental data. Point-by-point responses are typed in blue below.

Referee #1:

In this excellent small study, the authors use recently obtained structures of a plant ARGONAUTE protein as a basis to design experiments to obtain answers to a question of substantial importance for our understanding of the mechanistic basis of RNA interference phenomena: Why is it that base pairing between small RNA and target limited to the so-called seed region (nucleotides 2-7/8 of the small RNA) is sufficient for target regulation in metazoans, but not in plants? This question is all the more pertinent now that we know with certainty what was already expected from sequence conservation and Alphafold predictions: the overall structures of mammalian and plant Argonaute proteins are highly similar.

The authors first use rigorous experimentation to show directly that AGO10 - the plant AGO protein used for structural studies published elsewhere - binds with ~20-fold lower affinity compared to human Ago2 (Hs-Ago2) to a target RNA exhibiting seed complementarity. They then notice that a small loop in the Piwi domain adopts an ordered conformation in AGO10, but not in Hs-Ago2, including several interactions to the important helix-7 that is well-described in previous studies to influence seed pairing between guide and target RNA. The remainder of the paper uses, in the main, swapping experiments, in which the small Piwi-loop of Hs-Ago2 is inserted into AGO10, or the plant Piwi-loop (from AGO10 or AGO1) is inserted into Hs-Ago2, and the biochemical and/or silencing properties in cells are analysed. The experiments convincingly demonstrate that the major mechanistic basis of the different biochemical properties of plant and human miRNA-binding AGO proteins rely on the small Piwi-loop in the central cleft of the proteins. Specifically, the authors demonstrate that
(i) AGO10 harboring the Hs-Ago2 loop shows dramatically enhanced affinity to seed targets, including, importantly, a reduced dynamic range of binding affinity as a function of degree of small RNA:target complementarity
(ii) the majority of AGO10 molecules exhibit fast seed target-release kinetics, but in the presence of the Hs-Ago2 loop, the entire population switches to slow release.
(iii) Hs-Ago2 harboring the AGO10 loop has a structure with a well-defined conformation of the AGO10-loop, exhibits reduced affinity for seed targets, and, correspondingly, a dramatically enhanced dynamic range of binding affinity as a function of degree of small RNA:target complementarity
(iv) Hs-Ago2-AGO10 loop variant shows enhanced catalytic turnover in multi-turnover settings (but not in single-turnover conditions), consistent with more rapid target release
(v) the Hs-Ago2-AGO10 loop variant gives rise to substantially increased on-target/off-target ratios in experimental RNAi with siRNA duplexes and plasmid borne reporters in cell culture.

The experiments and well executed, results are surprisingly clear, and the interpretations of the results are well supported. More in vivo experiments, particularly on the plant side, would be desirable, but can be performed by other researchers following the publication of the results contained in this report. My suggestions for improvement of the current manuscript prior to publication are relatively minor, and focus on the crafting of the manuscript rather than the work itself that I find impressive and elegant.

> Thank you for this careful summary and your encouraging remarks.

(1) In the abstract and Introduction, I think the authors should take more care in how they describe the biological consequences of the clearly different properties in small RNA-guided targeting between plant and animal AGO proteins. First, it is not unequivocally established that "animals use miRNAs to sculpt the transcriptome" although some researchers advance this view. Please remember that although many mRNAs are targeted by a single miRNA in vivo, exactly what the biological consequence of this is, is more difficult to determine: many important effects of miRNAs in development can be explained by misregulation of only a few targets as demonstrated by multiple genetic studies in C. elegans and Drosophila, and there is good evidence that the multitude of conserved mRNA targets may serve to regulate miRNA activity rather than "sculpting the transcriptome" (Pinzon et al. (2016), Genome Research, https://genome.cshlp.org/content/early/2016/11/07/gr.205146.116).

> Thank you for raising this issue. We have included references to work from the Seitz lab and revised the abstract and introduction to present a more balanced view of current models for miRNA function in animals.

Similarly, the authors should note that to date, not a single study reporting on unbiased mapping of AGO1/miRNA binding sites throughout a plant transcriptome has been published, thus making it not fully established how miRNAs actually behave in vivo in plants.

> Thank you for raising this point. We added the following to the Introduction: "To our knowledge, unbiased mapping of AGO1/miRNA binding sites throughout a plant transcriptome has not been reported. Thus, the extent to which plant miRNAs interact with target mRNAs *in vivo* remains to be established. However, it is clear that the seed-based sites that function in animals do not reliably confer repression in plants (Liu *et al*, 2014)"

Notwithstanding the need to amend to oversimplification of some of these sections, the case of importance for the present study is clear enough: the targeting properties of animals and plant RISC are different!

(2) The literature used to support the statement that seed complementarity is not sufficient for miRNA targeting in plants should include the thorough in vivo work on this matter by the Axtell lab (Liu et al., (2014), Plant Cell, https://academic.oup.com/plcell/article/26/2/741/6097966).

> This is a perfect reference that we had totally missed. It has been added. Thank you.

(3) The treatment of AGO10 and AGO1 in the Introduction is not satisfactory. Although it is true that AGO10 is a close paralogue of the main miRISC component in plants, AGO1, it is not true to summarize their in vivo functions by the statement "with overlapping functions in vivo". This genetic interpretation is based on very early mutant studies that showed that ago1/ago10 double knockout mutants are embryonically lethal in contrast to either single mutant, although each of the single mutants has severe developmental defects in the genetic background analyzed at the time. We now know that ago1 shows these defects because of lack of miRNA-guided silencing, but ago10 can show defects because of enhanced AGO1-miR166 guided silencing. The "overlapping function" was a fair genetic interpretation in 1999, but is misleading to pass on to readers in 2022. Again, the authors need to take a bit more care not to

oversimplify which should be easily possible while maintaining the clear flow of the manuscript and emphasizing the most important points for their study.

> Thank you for pointing out this mistake. We removed our mention of the early genetic interpretation of Lynn *et al*, 1999. The only point we wanted to raise is AtAGO1 and AtAGO10 are close paralogs and thus may have similar biochemical and structural properties.

(4) The authors could consider re-labeling panels in Fig. 1 so that the close-ups become B and are referred to at first use. It is a bit hard to see the point of identical seed arrangement from the full structure views in A.

> We revised Fig. 1 as you suggested.

(5) The authors could consider including a comment on the E714H substitution comparing AGO10 to AGO1 in relation to the slightly behaviors of the insertions of these two proteins, in particular because interactions with E714 are highlighted.

> Thank you for raising this interesting point. We had not looked at this carefully, but indeed, the E714H substitution (comparing AGO10 to AGO1) suggests the AGO1 loop makes one less contact with the L2 domain and helix-7. This may explain why swapping the AGO1 loop into AGO10 slightly increased the affinity for a seed-matched target. We revised the Discussion to include: "Supporting this model, swapping the PIWI loop of AtAGO1 into AtAGO10, which we predict removes one contact modulating helix-7 (due to histidine in the place of E741 of AtAGO10) led to a small increase in affinity for a seed-matched target RNA (Fig. EV2A, B))."

(6) The authors could consider helping the reader with a separate illustration to guide the paragraph describing the "Structural model for miRNA targeting selectivity in plants". I found the reference to Fig. 1 a bit minimalistic in this regard.

> Thank you for this suggestion. The revised manuscript includes a cartoon schematic illustrating our proposed model (see Fig. EV3).

(7) The sentence "the N603 side chain is within H-bonding distance of the main chain amine of K355..." requires correction. Is it backbone amide of K355 or side chain amine?

> It is the mainchain amide of K355 (not amine). Correction made. Thanks so much.

(8) The literature used to support the statement that muliturnover rates are largely dictated by rate of product release is incomplete and should, as a minimum, also include the stop-flow studies reported by Deerberg et al. (2013) PNAS 110(44), 17850-5. https://www.pnas.org/doi/10.1073/pnas.1217838110

> Excellent point. We added the Deerberg reference, as well as Haley & Zamore 2004 and Rivas & Joshua-Tor 2005, who appear to have published the earliest data indicating product release is rate-limiting. Thank you.

(9) The discussion focuses very much on the, admittedly, very important result that selectivity and efficiency of RNAi in humans can be enhanced based on the findings in here. The results are also of high relevance for plant biology, however, and it would perhaps be relevant to discuss some of this?

> Thank you for this suggestion. After considering your comments and those of Referee #2 we decided to shift our primary focus away from increasing RNAi efficiency in humans and toward understanding functional differences between eukaryotic AGOs. The revised figure EV1 includes a comparison of the PIWI-loop sequences in diverse plants and animals. This comparison reveals that the clade 1 AGOs in Arabidopsis share a similar PIWI loop sequence that can be found in clade 1 AGOs from evolutionary distant members of the plant kingdom. By contrast, PIWI-loops of plant clade 2 and 3 AGOs are divergent from the clade 1 sequence. Although we do not yet know how the PIWI loops in clades 2 and 3 influence AGO behavior, we hypothesize that these variations may connect to the diversification of AGO function in plants. This idea is included in the revised Discussion.

One suggestion would be to include a comment on the fact that 15% of the AGO10 population shows slow release kinetics despite the presence of the Piwi-loop. Could one imagine that interaction with co-factors may increase this percentage such that seed-type matching could be regulated in plants, perhaps with ties to the many reports on translational repression by miRNAs that are highly complementary to their targets?

> We are also very interested in the idea that co-factors may regulate AGO behavior in plants. In fact, in a separate unpublished study (https://www.biorxiv.org/content/10.1101/2022.07.15.500266v1), we identified a plant-specific structural element in the AtAGO10 PIWI domain that may be a binding site for such co-factors. This element (termed the 'ß-finger', see Fig. 1A) is about 15 Å from the PIWI-loop and thus well-positioned to bind co-factors that regulate PIWI-loop behavior. However, we are hesitant to include these speculations in this manuscript because, to our knowledge, co-factors that alter AGO behavior have not yet been identified in any biological system.

(10) The language and writing style could benefit from a bit of improvement, although the manuscript reads fine overall.

> It is our hope that through the process of incorporating your suggestions and those of Referee #2, the manuscript has improved.

To sum up, I support publication of the manuscript without the need for further experimentation, but with some work put into improvement of the crafting of the manuscript. I would like to congratulate the authors for an interesting and well-executed study!

> Thank you very much!

Referee #2

The differences between animal and plant AGO proteins pose many important evolutionary, biological, and biochemical questions. This study solves a longstanding question in Argonaute enzymology: why do plant AGOs regulate their targets by cleavage ("siRNA-like) rather than the mechanism used by animals, in which AGOs bind a short, seed-matched sequence, then recruit other cellular components to reduce translation or stability of the target mRNA. The data are rigorous and clearly presented, and the manuscript is a pleasure to read. Even my major comments are fairly minor. I recommend publication of arevised manuscript that addresses my concerns below. (I would like to note that this study is more appropriate for EMBO J. than for EMBO Reports.)

> Thank you for these encouraging remarks.

Major Comments
(1) Page 7: How was kcat determined? For single-turnover reactions displaying burst kinetics, and the time-dependence of product formation corresponded to a pre-steady-state exponential burst (kburst = k2 + k3) followed by a linear steady-state phase, described by kcat, where kcat = k2 ´ k3/(k2 + k3). Please provide additional details for how both single- and multiple-turnover cleavage data was analyzed and whether E0 was fitted, measured via a binding assay, or assumed to be 100% active. Using this equation will allow the authors to determine for graphs like those in Figure S2D (e.g., bottom, gray line) the mix of burst and multiple-turnover rates that contribute to the apparent absence of a burst.

> Thank you for raising these questions and providing this advice. In the original manuscript, kcat was not determined, E0 was based on Bradford assay and assuming the protein was 100% active, and nearly all of our slicing data points were taken after >15% of the target RNA had been consumed, making it impossible to fit the burst-and-steady-state equation you provided.

In revising the manuscript, we were more rigorous and followed your advice carefully. We first determined the concentration of active enzyme in our HsAGO2 and HsAGO2+At-loop preparations by fitting preliminary room temperature (22 ˚C) slicing data to the burst-and-steady-state equation. We used 22˚C because we found that, compared to 37˚C, the 22˚C reactions proceeded more slowly and displayed greater differences between k2 and k3, which made our determinations of E0 more reliable. We then used the known concentrations of active enzyme to set up three independent slicing time course trials with [E0] = 5 nM and [target] = 100 nM at both 22˚C and 37˚C. Data points were taken rapidly enough to ensure that sufficient information for curve fitting was obtained before >15% of target RNA was consumed. Results, in revised Fig. 4D-E and Table 1, add further support to the conclusion that swapping the At-loop into HsAGO2 accelerates the cleavage of target RNAs under multiple turnover conditions by enabling product release.

(2) Is the 1.1–1.2-fold repression (green in Figure 5B) statistically different from no repression? If the error bars show standard deviation, they are surprisingly small. Was the error of the unrepressed control propagated through the error of the experiment using standard methods? For $z = c$*(x/y) error is propagated as Δz/z = [((Δx/x)^2) + ((Δy/y)^2)]^(1/2)

> Thank you for spotting this issue, which prompted us to better consider how we treat error. Indeed, as you guessed, we did not propagate the error correctly in the original submission. The data and figure have been corrected and show that the observed 1.1–1.2-fold repression is not significantly different from no repression.

(3) I understand the intent of the chart in Figure 5C, but from an evolutionary perspective, repression of the seed-matched reporter by the siRNA duplex or HsAGO2 RISC is on-target, not off-target. The siRNA is being used as a bona fide miRNA in an animal lacking an RNAi pathway. Perhaps a less normative nomenclature could be used, such as miRNA-like vs. siRNA-like repression?

> Yes, this makes perfect sense. We changed our nomenclature to siRNA-like vs. miRNA-like repression.

(4) An unpaired t-test can't formally be used to make pairwise comparisons in an experiment in which more than one hypothesis is tested (here, the hypotheses are is blue different from green and is red different from green). Typically, ANOVA followed by a post-hoc test is used.

> Thank you for pointing out this mistake. The revised figure has errors propagated correctly and was tested by one-way ANOVA, comparing the means of the siRNA duplex and HsAGO2-siRNA measurements to the mean of the HsAGO2+At-loop-siRNA measurement, followed by Dunnett's posthoc test. After error propagation and using the correct tests, we still observe a significant difference between silencing by HsAGO2-siRNA and HsAGO2+At-loop-siRNA, although the p-values are not as small as we had originally reported. Thank you.

(5) If it isn't too much work, it would be useful to know whether HsAGO2+At-loop shows superior target silencing compared to fly Ago2, which evolved to mediate RNAi and is rarely loaded with miRNAs in vivo.

> We would love to know the results of such a comparison. Unfortunately, despite efforts we have made on multiple occasions in the past, we have been unable to develop a system for preparing the DmAgo2-guide complex.

(6) Can phylogenetic analyses including other plant and animal AGOs help explain whether the animal or the plant PIWI loop is the likely ancestral state?

> I do not think so. We compared PIWI-loop sequences of other plant and animal AGOs and found the sequences vary widely, making the ancestral state unclear. However, within the plant or animal kingdoms, we did find correlations between PIWI-loop sequence conservation and specific AGO functions, particularly with respect to miRNAs.

Within Arabidopsis, the PIWI-loop sequences of plant clade 1 AGOs (AtAGO1, AtAGO5, and AtAGO10), which are responsible for most of the miRNA function in Arabidopsis, are similar to each other. Clade 1 AGOs from evolutionary distant members of the plant kingdom also have PIWI-loop sequences similar to the clade 1 AGOs in Arabidopsis. By contrast, PIWI-loops of Arabidopsis clades 2 and 3 AGOs are divergent from the clade 1 sequence. We hypothesize that variations in the PIWI-loop may connect to the diversification of AGO function in plants.

The PIWI-loop sequences in animal miRNA-class AGOs are distinct from any plant sequences but conserved between diverse members of the animal kingdom, even when comparing Cnidarian and Bilaterian animals. By contrast, the PIWI-loops of animal siRNA-class AGOs are

less stringently conserved. Even within arthropod siRNA-class AGOs, PIWI-loop sequences in insects are divergent from PIWI-loops in arachnids and the horseshoe crab.

We included our phylogenetic comparison of PIWI loops as Fig. EV1B in the revised manuscript.

(7) Could the more “rigid” loop in plant AGOs help them avoid cleaving targets with limited complementarity? Comparing AtAGO10-Hs and AtAGO10 cleavage pairing requirements could provide the answer.

> We are intrigued by this hypothesis because the AGO structural features that influence cleavage requirements are largely unknown. In fact, we are actively investigating the structural basis for siRNA function by HsAGO2. Part of this effort involves working to establish Cleave-’n-Seq in the lab. Once we have this technique established, we will be able to compare the cleavage preferences of HsAGO2, AtAGO10, AtAGO10-Hs, and HsAGO2-At. Thanks for the suggestion!

(8) Can the reduction in the range of affinities to just 4-fold upon introduction of Hs-loop into AtAGO10 be explained by a stronger affinity of AtAGO10-Hs for the canonical short seed and AtAGO10-Hs avoiding central pairing just like HsAGO2?

> Yes, partly. The greater affinity of AtAGO10-Hs for the seed raises the low end of the affinity range in this experiment, compressing the overall affinity range. The gains from central pairing are indeed reduced in the AtAGO10-Hs mutant, and curiously, affinity gains from extended pairing are also less. We combined the KD data into a single panel in the revised Fig. 2C to better illustrate this point. The main text now also explicitly states: “The smaller affinity range is the result of increased affinity for the seed region and decreased contributions from downstream guide-target pairing.”

Minor Comments
Abstract: (1) promiscuity implies lack of specificity; HsAGO2-bound miRNAs are highly specific, in that they bind perfect seed matches much more strongly than mismatched sites. There are simply more seed matches in the transcriptome because the site length is short compared to plant miRNAs. Sequence-specific RNA-binding proteins often recognize sites of the same length as a miRNA seed match.

> Yes, that makes sense. We removed phrases speaking to targeting ‘promiscuity’. We instead use the terms animal-like and plant-like miRNA targeting.

(2) Not all animals use miRNAs to repress hundreds of targets. See for example, Moran, Y. et al. Cnidarian microRNAs frequently regulate targets by cleavage. Genome Res 24, 651-663 (2014).

> We had totally missed this point. We changed the main text to read:
“The miRNA recognition elements (MREs) found in bilaterian target mRNAs consist of a core segment of complementarity to miRNA guide (g) nucleotides g2–g7 (counting from the miRNA 5' end), which is termed the miRNA seed region.”

Plant and animal AGOs are not more or less promiscuous, discerning, or stringent, they use different site lengths for target recognition. Moreover, the binding mechanisms of fly AGO2 and of animal PIWI proteins strongly suggest that HsAGO2 is more, not less, stringent than AGOs that recognize long target sites, in that it tolerates fewer mismatches in a single site. See also Herschlag, D. Implications of ribozyme kinetics for targeting the cleavage of specific RNA molecules in vivo: more isn't always better. Proc Natl Acad Sci U S A 88, 6921-6925 (1991).

> This makes sense. We have replaced the adjectives "promiscuous", "discerning", and "stringent" with miRNA-like and siRNA-like targeting, as indicated above.

Introduction: (1) The majority of the world's animals are arthropods, most of which likely use Ago2 (Ago1 is the arthropod homolog of AGO2) and siRNAs as an antiviral defense. It is worthwhile mentioning this (and commenting on the PIWI loop length in that protein).

> Thank you. Following this suggestion, and Major Comment (6) above, we included AGOs from diverse arthropods in our comparison of PIWI-loop sequences. The length of the DmAgo2 PIWI loop is the same as all other eukaryotic AGO sequences we examined. The DmAgo2 PIWI loop sequence is similar to other siRNA-class AGOs from selected insects, but this conservation was lost when we extended the comparison to members of the Chelicerata clade (spiders and horseshoe crabs). Fig. EV1B in the revised manuscript illustrates these observations. We also revised the introduction to mention that arthropods (and nematodes) possess siRNA-class AGOs that function as an antiviral defense.

Ago2 seems to be more like AGO2 than plant AGOs. Does this make structural sense?

>Yes, this does make a degree of sense with respect to our thoughts on the structural role of the PIWI-loop. As you showed previously (Wee, 2012), like HsAGO2, DmAgo2 creates a strong seed region, indicating that the DmAgo2 PIWI-loop does not dampen seed pairing. Indeed, the AlphaFold2 DmAgo2 prediction (https://alphafold.ebi.ac.uk/entry/Q9VUQ5) does not contain contacts between the PIWI and L2 loops similar to those that we associate with seed-dampening in the AtAGO10 and HsAGO2+At-loop structures. However, considering that we lack an experimentally validated structure of DmAGO2 and that there are considerable gross differences between HsAGO2 and DmAGO2 structure (i.e. the DmAGO2 lacks the 'Eukaryotic Insertion', which resides adjacent to the PIWI-loop and is proposed to modulate HsAGO2 target affinity (Bibel 2022, Golden 2017, and Quevillon Huberdeau 2017)), we are hesitant to include this line of reasoning in the manuscript.

This is especially important given the discussion about therapeutic siRNAs in animals lacking a formal RNAi pathway (top of page 3).

>That is an excellent point. We are hesitant to include speculations about structures that might make DmAGO2 specialized for RNAi, but we believe that it is worth noting biochemical similarities between DmAGO2 and the HsAGO2+At-loop mutant. The revised manuscript contains the following text: "Thus, the HsAGO2+At-loop mutant resembles *Drosophila* AGO2 (DmAGO2) (Wee et al., 2012), an enzyme responsible for binding and destroying viral and transposon transcripts, in that HsAGO2+At-loop binds far more tightly to fully complementary targets than to those matching only the seed."

(2) I think 2–3-fold is much greater than the typical effect of miRNA-binding on the steady-state abundance of an mRNA target for HsAGO2. If the typical effect were that large, no one would need to use CDF plots to see it.

> This is an interesting conundrum. We examined data from Baek *et al*, 2008 and found that, as you indicated, the average effect reported is less than 2-fold. In fact, figure 1 shows that transfection of miR-124 into HeLa cells led to, on average, a mere ~15% reduction in the expression of genes with one or more conserved 8mer matches to miR-124. This mild effect resonates with Seitz's question of whether most of these genes should be considered physiologically relevant targets of repression. We, therefore, decided to look back at miRNA-target interactions with well-validated physiological contributions, which led us to the work of Bagga *et al*, 2005, who reported that *let-7* represses *lin-41* ~3-fold between the L2 and L3 stages of *C. elegans*. Taking all this information into consideration, we decided to change the text to read: "miRNA target recognition leads to a moderate (1.1–3 fold) reduction in gene expression".

(3) There is a growing body of evidence that animal miRNAs do not sculpt the genome. Both views should be presented. E.g., Pinzón, N. et al. microRNA target prediction programs predict many false positives. Genome Res 27, 234-245 (2017) and Seitz, H. Issues in current microRNA target identification methods. RNA Biol 14, 831-834 (2017).

> Thank you for raising this point. These references have been added and the revised abstract and introduction have a more balanced presentation of these two models.

(4) What clinical trial has failed as a consequence of miRNA-like off-target effects? To the best of my knowledge, it is not possible to achieve a sufficiently high intracellular concentration of drug in RISC in the target tissue to mediate such effects. (I doubt that there would be five FDAapproved siRNA drugs in clinical use today if such effects really happened.)

> We are happy to receive the best of your knowledge here! Upon rereading the cited papers, we found that we were in error about failed clinical trials. We have removed the statement and toned down the language surrounding mitigating miRNA-like off-target effects in therapeutic settings. Thank you for pointing this out.

Results: (1) When percent active enzyme is used in calculating dissociation constants, animal RISCs whose seed sequence fully complementary to a target site have KD values in the pM, not nM range. E.g., this manuscript; Jouravleva et al. (Cell Reports Methods 2022); Salomon et al. (Cell 2015); Becker et al. (Mol Cell 2019).

> We reviewed the references cited above and found that, as you indicated, seed-matched target sites indeed have KD values in the pM range for most seed sequences. Thank you. We found the data in Jouravleva et al. to be particularly helpful because affinities of multiple different seed sequences were measured side-by-side. Borrowing terminology from Jouravleva et al., we changed the main text to read: "dissociation constants typically in the subnanomolar range". We included 'typically' here because of miR-21, which appears to be an outlier. If we are interpreting the data in Salomon et al. correctly, the KD for miR-21 is 13 ± 0.2 nM (calculated

from the ratio of reported koff / kon values). This number closely matches the miR-21 KD value of 21 ± 1 nM reported by Ziv et al., Nature Methods, 2018 (Supplementary Figure 12h).

Figure 1B: it would be helpful to include the guide and target sequences in the figure.

> We added guide and target sequences to the figure.

Figure 4D, right panel: should K (equilibrium constant) be k (rate constant)?

> Yes, thank you (although we ended up replacing this panel with new data in response to Major Comment #1).

Also, should the callout " Fig. 4E,F" on page 7 be 4D?

> Yes. See above. Thanks.

Wee et al. (Cell 2012) used mouse, not human AGO2 (they are nearly identical, of course).

> Yes, very true.

Thermus thermophilus TtAgo behaves like an animal AGO at 37°C and like a plant AGO at 55°C (Smith, C. S. et al. An automated Bayesian pipeline for rapid analysis of single-molecule binding data. Nat Commun 10, 272 (2019). Can your observations help explain why?

> I don't know. In our model, AtAGO10 dampens the seed by using its PIWI-loop to hold the protein in a 'closed' conformation with restricted access to g6–g8. We have not examined the effects of temperature on seed-pairing, but we imagine that higher temperature would increase conformational dynamics and thereby compromise the ability of the PIWI-loop to hold the 'closed' conformation, leading to a more exposed, and therefore undampened, seed.

Like HsAGO2 and AtAGO10, the guide-only crystal structure of TtAGO (PDB 3DLH) adopts a 'closed' conformation with restricted access to g6–g8. Therefore, considering protein structure alone, we would predict that increasing temperature, which we assume would increase conformational dynamics and access to the seed, would enable the seed-pairing, not make it weaker.

We suggest that the dwell-time temperature dependence of TtAGO on seed-only targets may be largely due to the propensity of shorter nucleic acid duplexes to melt more readily at elevated temperatures than longer duplexes (Cantor and Schimmel (1980). Chapter 23: Statistical Mechanics and Kinetics of Nucleic Acid Interactions. In *Biophysical Chemistry. Part III: The behavior of biological macromolecules*). The HsAGO2-guide complex is relatively stable at 55˚C (PMID: 28939659, Fig. EV1B,C). Perhaps HsAGO2 would also behave like a plant AGO at this temperature?

Perhaps the title is over broad given that only two AGO proteins were studied (HsAGO2 and AtAGO10)?

> That is a fair point. We have changed the title to: “A tiny loop in the Argonaute PIWI domain tunes small RNA seed strength”. This more precisely reflects the main findings.

1st Revision - Editorial Decision 29th Mar 2023

Dear Ian,

Thank you for the submission of your revised manuscript. We have now received the enclosed report from the referee who was asked to assess it, and I am happy to say that the referee supports its publication now.

Only a few editorial requests will need to be addressed:

- The manuscript has 5 main figures but the results and discussion sections are not combined. Please either add one more main figure, or combine the results and discussion sections to publish your study as a short report. You can find more info about our article types in our guide to authors.

- Please add up to 5 keywords to the ms file.

- Please remove the authors credits from the ms file. We now use CRediT to specify the contributions of each author in the journal submission system. CRediT replaces the author contribution section. Please use the free text box to provide more detailed descriptions, if you wish. See also guide to authors https://www.embopress.org/page/journal/14693178/authorguide#authorshipguidelines.

- Fig 4E callout is missing, please add.

- Please add the subheading 'Expanded View Figure Legends'

- Tables EV1-EV3 should be uploaded individually (as word or excel files) using the file type Expanded View. Please remove them from the ms file.

- I attach to this email a related ms file with comments by our data editors. Please address all comments in the final ms.

EMBO press papers are accompanied online by A) a short (1-2 sentences) summary of the findings and their significance, B) 2-3 bullet points highlighting key results and C) a synopsis image that is exactly 550 pixels wide and 200-600 pixels high (the height is variable). You can either show a model or key data in the synopsis image. Please note that text needs to be readable at the final size. Please send us this information along with the final manuscript.

I look forward to seeing a final version of your manuscript as soon as possible. Please use this link to submit your revision: https://embor.msubmit.net/cgi-bin/main.plex

Best wishes,
Esther

Esther Schnapp, PhD
Senior Editor
EMBO reports

Referee #2:

The authors have done a superb job revising the manuscript. It should be published without delay.

The authors have addressed all minor editorial requests.

2nd Revision - Editorial Decision 5th Apr 2023

Prof. Ian MacRae
The Scripps Research Institute
Department of Integrative Structural and Computational Biology
MB-45
10550 N. Torrey Pines Road
La Jolla, CA 92037
United States

Dear Ian,

I am very pleased to accept your manuscript for publication in the next available issue of EMBO reports. Thank you for your contribution to our journal.

At the end of this email I include important information about how to proceed. Please ensure that you take the time to read the information and complete and return the necessary forms to allow us to publish your manuscript as quickly as possible.

As part of the EMBO publication's Transparent Editorial Process, EMBO reports publishes online a Review Process File to accompany accepted manuscripts. As you are aware, this File will be published in conjunction with your paper and will include the referee reports, your point-by-point response and all pertinent correspondence relating to the manuscript.

If you do NOT want this File to be published, please inform the editorial office within 2 days, if you have not done so already, otherwise the File will be published by default [contact: emboreports@embo.org]. If you do opt out, the Review Process File link will point to the following statement: "No Review Process File is available with this article, as the authors have chosen not to make the review process public in this case."

Thank you again for your contribution to EMBO reports and congratulations on a successful publication. Please consider us again in the future for your most exciting work.

Best regards,
Esther

Esther Schnapp, PhD
Senior Editor
EMBO reports

****

THINGS TO DO NOW:

Please note that you will be contacted by Wiley Author Services to complete licensing and payment information. The required 'Page Charges Authorization Form' is available here: https://www.embopress.org/pb-assets/embo-site/er_apc.pdf - please download and complete the form and return to embopressproduction@wiley.com

EMBO Press participates in many Publish and Read agreements that allow authors to publish Open Access with reduced/no publication charges. Check your eligibility: https://authorservices.wiley.com/author-resources/Journal-Authors/open-access/affiliation-policies-payments/index.html

You will receive proofs by e-mail approximately 2-3 weeks after all relevant files have been sent to our Production Office; you should return your corrections within 2 days of receiving the proofs.

Please inform us if there is likely to be any difficulty in reaching you at the above address at that time. Failure to meet our deadlines may result in a delay of publication, or publication without your corrections.

All further communications concerning your paper should quote reference number EMBOR-2022-55806V3 and be addressed to emboreports@wiley.com.

Should you be planning a Press Release on your article, please get in contact with emboreports@wiley.com as early as possible, in order to coordinate publication and release dates.

EMBO Press Author Checklist

Corresponding Author Name: Ian J. MacRae	
Journal Submitted to: EMBO Reports	
Manuscript Number: EMBOR-2022-55806V1	

USEFUL LINKS FOR COMPLETING THIS FORM
The EMBO Journal - Author Guidelines
EMBO Reports - Author Guidelines
Molecular Systems Biology - Author Guidelines
EMBO Molecular Medicine - Author Guidelines

Reporting Checklist for Life Science Articles (updated January 2022)
This checklist is adapted from Materials Design Analysis Reporting (MDAR) Checklist for Authors. MDAR establishes a minimum set of requirements in transparent reporting in the life sciences (see Statement of Task: 10.31222/osf.io/9sm4x). Please follow the journal's guidelines in preparing your manuscript.
Please note that a copy of this checklist will be published alongside your article.

Abridged guidelines for figures
1. Data
The data shown in figures should satisfy the following conditions:
- the data were obtained and processed according to the field's best practice and are presented to reflect the results of the experiments in an accurate and unbiased manner.
- ideally, figure panels should include only measurements that are directly comparable to each other and obtained with the same assay.
- plots include clearly labeled error bars for independent experiments and sample sizes. Unless justified, error bars should not be shown for technical replicates.
- if n<5, the individual data points from each experiment should be plotted. Any statistical test employed should be justified.
- Source Data should be included to report the data underlying figures according to the guidelines set out in the authorship guidelines on Data Presentation.

2. Captions
Each figure caption should contain the following information, for each panel where they are relevant:
- a specification of the experimental system investigated (eg cell line, species name).
- the assay(s) and method(s) used to carry out the reported observations and measurements.
- an explicit mention of the biological and chemical entity(ies) that are being measured.
- an explicit mention of the biological and chemical entity(ies) that are altered/varied/perturbed in a controlled manner.
- the exact sample size (n) for each experimental group/condition, given as a number, not a range;
- a description of the sample collection allowing the reader to understand whether the samples represent technical or biological replicates (including how many animals, litters, cultures, etc.).
- a statement of how many times the experiment shown was independently replicated in the laboratory.
- definitions of statistical methods and measures:
  - common tests, such as t-test (please specify whether paired vs. unpaired), simple χ^2 tests, Wilcoxon and Mann-Whitney tests, can be unambiguously identified by name only, but more complex techniques should be described in the methods section;
  - are tests one-sided or two-sided?
  - are there adjustments for multiple comparisons?
  - exact statistical test results, e.g., P values = x but not P values < x;
  - definition of 'center values' as median or average;
  - definition of error bars as s.d. or s.e.m.

Please complete ALL of the questions below.
Select "Not Applicable" only when the requested information is not relevant for your study.

Materials

Newly Created Materials	Information included in the manuscript?	In which section is the information available? (Reagents and Tools Table, Materials and Methods, Figures, Data Availability Section)
New materials and reagents need to be available; do any restrictions apply?	Not Applicable	

Antibodies	Information included in the manuscript?	In which section is the information available? (Reagents and Tools Table, Materials and Methods, Figures, Data Availability Section)
For **antibodies** provide the following information:<br>- Commercial antibodies: RRID (if possible) or supplier name, catalogue number and or/clone number<br>- Non-commercial: RRID or citation	Not Applicable	

DNA and RNA sequences	Information included in the manuscript?	In which section is the information available? (Reagents and Tools Table, Materials and Methods, Figures, Data Availability Section)
**Short novel DNA or RNA including primers, probes**: provide the sequences.	Yes	Table EV2

Cell materials	Information included in the manuscript?	In which section is the information available? (Reagents and Tools Table, Materials and Methods, Figures, Data Availability Section)
**Cell lines:** Provide species information, strain. Provide accession number in repository **OR** supplier name, catalog number, clone number, and/**OR** RRID.	Yes	Materials and Methods
**Primary cultures:** Provide species, strain, sex of origin, genetic modification status.	Not Applicable	
Report if the cell lines were recently **authenticated** (e.g., by STR profiling) and tested for mycoplasma contamination.	Not Applicable	

Experimental animals	Information included in the manuscript?	In which section is the information available? (Reagents and Tools Table, Materials and Methods, Figures, Data Availability Section)
**Laboratory animals or Model organisms:** Provide species, strain, sex, age, genetic modification status. Provide accession number in repository **OR** supplier name, catalog number, clone number, **OR** RRID.	Not Applicable	
**Animal observed in or captured from the field:** Provide species, sex, and age where possible.	Not Applicable	
Please detail **housing and husbandry conditions**.	Not Applicable	

Plants and microbes	Information included in the manuscript?	In which section is the information available? (Reagents and Tools Table, Materials and Methods, Figures, Data Availability Section)
**Plants:** provide species and strain, ecotype and cultivar where relevant, unique accession number if available, and source (including location for collected wild specimens).	Not Applicable	
**Microbes:** provide species and strain, unique accession number if available, and source.	Yes	Materials and Methods

Human research participants	Information included in the manuscript?	In which section is the information available? (Reagents and Tools Table, Materials and Methods, Figures, Data Availability Section)
If collected and within the bounds of privacy constraints report on age, sex and gender or ethnicity for all study participants.	Not Applicable	

Core facilities	Information included in the manuscript?	In which section is the information available? (Reagents and Tools Table, Materials and Methods, Figures, Data Availability Section)
If your work benefited from core facilities, was their service mentioned in the acknowledgments section?	Not Applicable	

Design

Study protocol	Information included in the manuscript?	In which section is the information available? (Reagents and Tools Table, Materials and Methods, Figures, Data Availability Section)
If study protocol has been **pre-registered, provide DOI in the manuscript**. For clinical trials, provide the trial registration number **OR** cite DOI.	Not Applicable	
Report the **clinical trial registration number** (at ClinicalTrials.gov or equivalent), where applicable.	Not Applicable	

Laboratory protocol	Information included in the manuscript?	In which section is the information available? (Reagents and Tools Table, Materials and Methods, Figures, Data Availability Section)
Provide DOI OR other citation details if **external detailed step-by-step protocols** are available.	Not Applicable	

Experimental study design and statistics	Information included in the manuscript?	In which section is the information available? (Reagents and Tools Table, Materials and Methods, Figures, Data Availability Section)
Include a statement about **sample size** estimate even if no statistical methods were used.	Yes	Figure Legends
Were any steps taken to minimize the effects of subjective bias when allocating animals/samples to treatment (e.g. **randomization procedure**)? If yes, have they been described?	Not Applicable	
Include a statement about **blinding** even if no blinding was done.	Yes	Materials and Methods
Describe **inclusion/exclusion criteria** if samples or animals were excluded from the analysis. Were the criteria pre-established?<br><br>If sample or data points were omitted from analysis, report if this was due to attrition or intentional exclusion and provide justification.	Yes	Figure Legends
For every figure, are **statistical tests** justified as appropriate? Do the data meet the assumptions of the tests (e.g., normal distribution)? Describe any methods used to assess it. Is there an estimate of variation within each group of data? Is the variance similar between the groups that are being statistically compared?	Yes	Figure Legends

Sample definition and in-laboratory replication	Information included in the manuscript?	In which section is the information available? (Reagents and Tools Table, Materials and Methods, Figures, Data Availability Section)
In the figure legends: state number of times the experiment was **replicated** in laboratory.	Yes	
In the figure legends: define whether data describe **technical or biological replicates**.	Yes	

Ethics

Ethics	Information included in the manuscript?	In which section is the information available? (Reagents and Tools Table, Materials and Methods, Figures, Data Availability Section)
Studies involving **human participants**: State details of **authority granting ethics approval** (IRB or equivalent committee(s), provide reference number for approval.	Not Applicable	
Studies involving **human participants**: Include a statement confirming that **informed consent** was obtained from all subjects and that the experiments conformed to the principles set out in the WMA Declaration of Helsinki and the Department of Health and Human Services Belmont Report.	Not Applicable	
Studies involving **human participants:** For publication of **patient photos**, include a statement confirming that consent to publish was obtained.	Not Applicable	
Studies involving experimental **animals**: State details of **authority granting ethics approva**l (IRB or equivalent committee(s), provide reference number for approval. Include a statement of compliance with ethical regulations.	Not Applicable	
Studies involving **specimen and field samples:** State if relevant **permits** obtained, provide details of authority approving study; if none were required, explain why.	Not Applicable	

Dual Use Research of Concern (DURC)	Information included in the manuscript?	In which section is the information available? (Reagents and Tools Table, Materials and Methods, Figures, Data Availability Section)
Could your study fall under dual use research restrictions? Please check biosecurity documents and list of **select agents and toxins** (CDC): https://www.selectagents.gov/sat/list.htm	Not Applicable	
If you used a select agent, is the security level of the lab appropriate and reported in the manuscript?	Not Applicable	
If a study is subject to dual use research of concern regulations, is the name of the **authority granting approval and reference number** for the regulatory approval provided in the manuscript?	Not Applicable	

Reporting

The MDAR framework recommends adoption of discipline-specific guidelines, established and endorsed through community initiatives. Journals have their own policy about requiring specific guidelines and recommendations to complement MDAR.

Adherence to community standards	Information included in the manuscript?	In which section is the information available? (Reagents and Tools Table, Materials and Methods, Figures, Data Availability Section)
State if relevant guidelines or checklists (e.g., **ICMJE, MIBBI, ARRIVE, PRISMA**) have been followed or provided.	Not Applicable	
For **tumor marker prognostic studies,** we recommend that you follow the **REMARK** reporting guidelines (see link list at top right). See author guidelines, under 'Reporting Guidelines'. Please confirm you have followed these guidelines.	Not Applicable	
For **phase II and III randomized controlled trials**, please refer to the **CONSORT** flow diagram (see link list at top right) and submit the CONSORT checklist (see link list at top right) with your submission. See author guidelines, under 'Reporting Guidelines'. Please confirm you have submitted this list.	Not Applicable	

Data Availability

Data availability	Information included in the manuscript?	In which section is the information available? (Reagents and Tools Table, Materials and Methods, Figures, Data Availability Section)
Have **primary datasets** been deposited according to the journal's guidelines (see 'Data Deposition' section) and the respective accession numbers provided in the Data Availability Section?	Yes	Data Availability
Were **human clinical and genomic datasets** deposited in a public access-controlled repository in accordance to ethical obligations to the patients and to the applicable consent agreement?	Not Applicable	
Are **computational models** that are central and integral to a study available without restrictions in a machine-readable form? Were the relevant accession numbers or links provided?	Not Applicable	
If publicly available data were reused, provide the respective **data citations in the reference list**.	Not Applicable	
